# Proteogenomic analysis of human cerebrospinal fluid identifies neurologically relevant regulation and informs causal proteins for Alzheimer’s disease

**DOI:** 10.21203/rs.3.rs-2814616/v1

**Published:** 2023-06-09

**Authors:** Carlos Cruchaga, Dan Western, Jigyasha Timsina, Lihua Wang, Ciyang Wang, Chengran Yang, Muhammad Ali, Aleksandra Beric, Priyanka Gorijala, Patsy Kohlfeld, John Budde, Allan Levey, John Morris, Richard Perrin, Agustín Ruiz, Marta Marquié, Mercè Boada, Itziar de Rojas, Jarod Rutledge, Hamilton Oh, Edward Wilson, Yann Le Guen, Ignacio Alvarez, Miquel Aguilar, Michael Greicius, Pau Pastor, David Pulford, Laura Ibanez, Tony Wyss-Coray, Yun Ju Sung, Bridget Phillips

**Affiliations:** Washington University School of Medicine; Department of Psychiatry, Washington University School of Medicine, St. Louis, MO, USA; Department of Psychiatry, Washington University School of Medicine, St. Louis, MO, USA; Washington University School of Medicine; Washington University School of Medicine; Washington University in St. Louis; Washington University School of Medicine; Washington University School of Medicine; Washington University School of Medicine; Washington University School of Medicine, St Louis, MO, USA; Emory; Knight Alzheimer Disease Research Center; Washington University in St. Louis; Fundació ACE; Universitat Internacional de Catalunya; Memory Clinic of Fundaciò ACE, Catalan Institute of Applied Neurosciences; Universitat Internacional de Catalunya; Stanford University; Stanford University; Stanford University; Stanford University; Fundació Docència i Recerca Mútua Terrassa, Terrassa, Barcelona, Spain; University Hospital Mutua Terrassa; Stanford School of Medicine; University Hospital Germans Trias i Pujol; GlaxoSmithKline; Washington University in St. Louis; Stanford University; Washington University in St. Louis; Washington University in St. Louis

## Abstract

The integration of quantitative trait loci (QTL) with disease genome-wide association studies (GWAS) has proven successful at prioritizing candidate genes at disease-associated loci. QTL mapping has mainly been focused on multi-tissue expression QTL or plasma protein QTL (pQTL). Here we generated the largest-to-date cerebrospinal fluid (CSF) pQTL atlas by analyzing 7,028 proteins in 3,107 samples. We identified 3,373 independent study-wide associations for 1,961 proteins, including 2,448 novel pQTLs of which 1,585 are unique to CSF, demonstrating unique genetic regulation of the CSF proteome. In addition to the established chr6p22.2-21.32 HLA region, we identified pleiotropic regions on chr3q28 near *OSTN* and chr19q13.32 near *APOE* that were enriched for neuron-specificity and neurological development. We also integrated this pQTL atlas with the latest Alzheimer’s disease (AD) GWAS through PWAS, colocalization and Mendelian Randomization and identified 42 putative causal proteins for AD, 15 of which have drugs available. Finally, we developed a proteomics-based risk score for AD that outperforms genetics-based polygenic risk scores. These findings will be instrumental to further understand the biology and identify causal and druggable proteins for brain and neurological traits.

Genome-wide association studies (GWAS) have burst onto the genetic landscape over the last 15 years. Many traits and common diseases have been studied in hundreds of thousands of individuals, identifying genetic loci associated with height, cancer, coronary artery disease, and many other traits^1–3^. However, translation of the associations identified by GWAS is often challenging, as many relevant loci fall in generich or intergenic regions. These associations make interpretation and assignment of loci to specific genes difficult.

Recently, large databases analyzing the genetic regulation of biological molecules, including the GTEx consortium, eQTLGen, and MetaBrain^4–6^, have described genetic loci affecting mRNA levels. These resources have been integrated with disease GWAS analyses to prioritize functional genes at previously uncharacterized loci^7^. However, analyses focusing on mRNA miss disease-relevant biology. First, many of the studies focus solely on genetic variants that are in close proximity to the gene encoding the mRNA, preventing the identification of cross-genome regulatory effects. Second, the correlation between levels of mRNA and their encoded proteins is weak^8,9^, while proteins are typically the molecule that most-directly act on disease. As a result of this low correlation, the overlap between expression (mRNA) quantitative trait loci (QTL) and protein QTL is also low, suggesting genetic regulation unique to proteins^10^.

Large studies have investigated genetic association of protein levels, but they have overwhelmingly focused on plasma^11–15^; previous work has shown that plasma proteogenomics shares little overlap with the brain^16^. Small studies have analyzed the proteogenomic signature of cerebrospinal fluid (CSF)^16–20^. Targeting specific CSF proteins has proven successful at elucidating causal genes at some of the disease GWAS loci, including an association for the TREM2 protein at the *MS4A* locus providing a potential mechanism about how *MS4A* modifies AD risk^7^. However, these studies are limited by comparatively small sample sizes or number of proteins analyzed.

Alzheimer’s disease (AD) is the most common highly heritable neurodegenerative disease, with heritability estimates in the range of 60 to 80%^21^. GWAS have identified over 75 genetic loci associated with AD^22^. However, the functional genes driving the associations for most of these loci are still unknown, or how these GWAS genes interact between them in specific pathways and disease mechanisms.

Here, we present the largest-to-date investigation of the genomic signature of the human CSF proteome, in both number of proteins and sample size. We analyze the genetic regulation of 7,028 unique proteins using CSF from 3,107 healthy and neurologically impaired individuals. We classify proteogenomic hotspots that regulate multiple proteins, then integrate our results with a large-scale AD risk GWAS to identify novel causal and druggable proteins relevant to AD. Finally, we use our AD-associated proteins to build a prediction model that excels at classifying AD biomarker status.

## Unique genetic architecture of CSF proteomics

**Figure 1:**
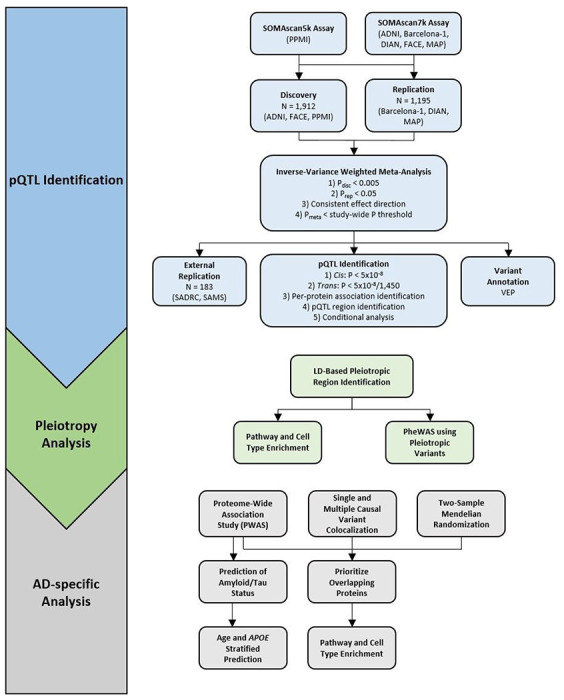
Study Design. PPMI: Parkinson’s Progression Markers Initiative; ADNI: Alzheimer’s Disease Neuroimaging Inititiative; DIAN: Dominantly Inherited Alzheimer’s Network; FACE: Fundació Ace; MAP: Knight-ADRC Memory and Aging Project.

**Figure 2:**
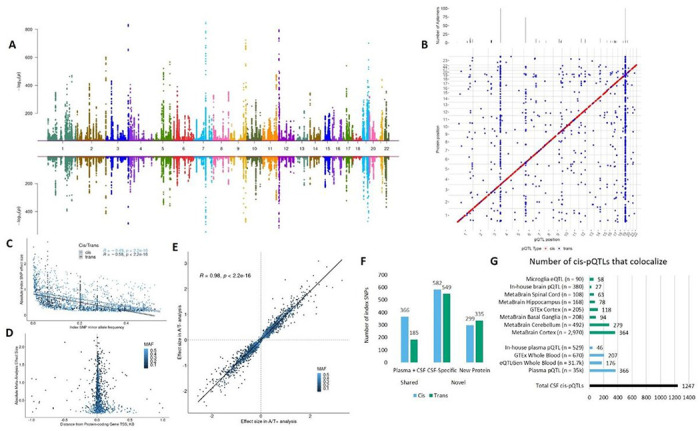
Cerebrospinal fluid pQTLs are consistent across disease and largely tissue and molecule-specific. **A.** Miami plot of combined results for each aptamer from pQTL analysis in the discovery (top, 1,912 samples and 7,559 aptamers) and replication (bottom, 1,195 samples and 7,028 aptamers) datasets. The x-axis represents genome position of an associated variant and the y-axis represent the −log10(p-value) for association of each SNP with an aptamer. **B.** 2D Manhattan plot of 2,316 index pQTLs for 1,961 aptamers that were significant after meta-analysis. The x-axis position represents the genome position of the pQTL signal. The y-axis position represents the location of the protein-coding gene corresponding to the aptamer with a pQTL. Color represents *cis* or *trans* status of the index SNP (cis, red; trans, blue). The top panel maps the pleiotropic regions of the genome (limited to 100 aptamers with an association in the same region). **C.** Scatter plot of index pQTL SNP effect size (y-axis) vs index pQTL SNP minor allele frequency (x-axis). Color represents *cis* or *trans* status of the index SNP (*cis*, blue; *trans*, black). Correlation was calculated using the Pearson method. **D.** Scatter plot of *cis* index pQTL effect sizes (y-axis) vs distance from the transcription start site of the gene encoding the associated protein (x-axis). Color represents the minor allele frequency (darker = less common). **E.** Scatter plot of effect size of index pQTL variants in dichotomized amyloid/tau positive samples (x-axis) vs dichotomized amyloid/tau negative samples (y-axis). Color represents minor allele frequency (darker = less common). Correlation was calculated using the Pearson method. **F.** Colocalization of CSF pQTL associations with plasma pQTL associations from Ferkingstad et al^11^. Plasma + CSF represents pQTL associations that colocalized across tissues. CSF-specific represents pQTL associations that did not colocalize with plasma pQTLs for the same protein. New Proteins are pQTL associations for proteins measured in CSF but not plasma. Color represents *cis* or *trans* status of the index CSF pQTL (*cis*, blue; *trans*, green). **G.** Colocalization of *cis* CSF pQTL associations with various QTL types. Green bars represent brain-relevant tissues, while blue bars represent other tissues. Bar labels represent the number of colocalizing QTLs.

We performed the proteogenomic analysis of CSF by integrating proteomics (aptamer-based assay: SOMAscan 7k^23,24^; Supp Fig. 1; Supp. Tables S1&S2) and genomic data in a total of 3,107 unrelated Non-Hispanic White (NHW) individuals (Supp Fig. 2). We utilized a three-stage study design (Fig. 1): 1) a discovery stage including 1,912 CSF samples and genomic data from unrelated NHW individuals from the Alzheimer’s Disease Neuroimaging Initiative (ADNI), Parkinson’s Progression Marker Initiative (PPMI), and Ace Alzheimer Center Barcelona (FACE) with 7,559 aptamers passing stringent QC, corresponding to 6,334 unique proteins (Fig. 2A, top; Supp. Figure 3; see Methods for proteomic data generation and QC); 2) a replication stage including 1,195 CSF samples and genomic data from unrelated NHW individuals from the Knight-ADRC Memory and Aging Project (Knight-ADRC),the Dominantly-Inherited Alzheimer’s Network (DIAN), and Barcelona-1 with 7,028 aptamers passing QC, corresponding to 6,177 unique proteins (Fig. 2A, Supp. Figure 4, Supp. Table S1&S2); and 3) a fixed-effect meta-analysis of the aptamers measured in both discovery and replication (Table 1). For pre-QC descriptions of the proteomics samples and samples removed by each step, see Supp. Tables S3&S4. In the meta-analysis, we considered protein-variant associations (pQTL) to be significant if they had P_disc_ < 0.005, P_rep_ < 0.05, a consistent direction of effect in discovery and replication, and study-wide significant p-value in the meta-analysis (P < 5×10^− 8^ for *cis* variants and P < 3.45×10^− 11^ for *trans* variants; see materials and methods).

In the meta-analysis, we identified 2,316 index pQTL associations for 1,961 protein aptamers (1,786 unique proteins; Fig. 2B, Supp. Table S5). Of those, 1,247 (53.8%) were *cis* (+/− 1MB from TSS) and 1,069 (46.2%) were *trans*-pQTL. Correlation of effect sizes for the study-wide significant variants was extremely high (*R* = 0.98, P < 2.2×10^− 16^, Supp. Figure 5, Supp. Table S5), confirming that both cohorts are contributing to the associations observed and highlighting the robustness of these pQTL associations. We further replicated our findings using a third independent cohort with SOMAscan proteomics measurements and genomic data (N = 183 from the Stanford Iqbal Farrukh and Asad Jamal Alzheimer’s Disease Research Center). We observed a strong index pQTL variant effect size correlation between the meta-analysis and external replication (1,676 variants tested) when comparing the SOMAscan results (N_rep_ = 183, *R^2^* = 0.92, P < 2.2×10^− 16^, Supp. Figure 6, Supp. Table S5). For pathway enrichment analysis results for all proteins with a pQTL, see Supp. Figure 9 and Supp. Table S6.

Because a large portion of our dataset (2,031, 65.4%, Table 1) is derived from individuals with Alzheimer’s disease, we sought to determine if our associations were consistent across healthy and affected individuals. We classified individuals into AD-relevant biomarker groups using the amyloid/tau/neurodegeneration (A/T/N) framework^25^ (see methods). We performed association analysis for our index pQTL variants stratifying by AD biomarker positive (A^+^T^+^; N = 775) vs negative (A^−^T^−^; N = 889). When comparing effect sizes of the index pQTL variants, we observed a strong correlation between A^−^T^−^ vs A^+^T^+^ (*R* = 0.98, P < 2.2×10^− 16^; Fig. 2E, Supp. Table S5), meta-analysis vs A^+^T^+^ (*R* = 0.98, P < 2.2×10^− 16^, Supp. Figure 7A), and meta-analysis vs A^−^T^−^ (*R* = 0.99, P < 2.2×10^− 16^, Supp. Figure 7B), indicating that the pQTL identified in our meta-analyses are consistent across disease states. However, we did observe some associations that were biased toward either diseased or healthy individuals (see Extended Results).

GWAS loci can often have independent significant variants in the same locus^26^. To identify independent signals in the same locus, we performed conditional analyses using GCTA-COJO^27^ (Supp. Figure 5, Supp. Table S7). In total, we identified 3,373 conditionally independent associations (see Extended Results, Supp. Table S7). Most aptamers (1,137, 58.0%) had a single independent association, but 824 aptamers had at least two, 337 had at least three, 144 had at least four, and one protein (GSTM1) had 11 independent associations at its *cis* locus. This suggests that many proteins have complex mechanisms regulating their CSF levels. These analyses were performed at a study-wide level, so it is possible that we excluded many additional independent associations below our study-wide significance threshold. We also used VEP to annotate each conditionally independent association.

We identified all variants in linkage disequilibrium (LD, *R^2^* > 0.8) with each conditionally independent pQTL variant and performed variant annotation on the associations using the Ensembl Variant Effect Predictor (VEP)^28^, determining the most severe consequence corresponding to each variant set (*R*^2^ > 0.8, Supp. Table S8&S9). The largest proportion of the variants were intronic (N = 1,156, 34.3%), followed by upstream/downstream of nearby genes (691, 20.5%), missense (799, 23.7%), or intergenic (324, 9.6%, Supp. Figure 8, Supp. Table S7). In order to determine if the pQTL were enriched in any specific annotation category, we compared our proportions to those from VEP annotation of 3,373 random variants and 1,000 permutations. We observed a significant enrichment in multiple categories of protein-altering variants, including missense, splice donor, and protein-truncating variants (see Extended Results, Supp. Figure 8D, Supp. Table S10), but not intronic, intergenic, or non-coding. While intronic variants were the most prevalent pQTL annotation overall, the percentage annotated as intronic (34.3%) was much lower than the randomly selected variants (51.7% intronic on average). The independent variants were also enriched for protein-altering variants overall (P < 0.001), indicating that pQTL are enriched for coding variants in comparison with other variant types.

Common variants in both *cis* and *trans* had lower effect sizes on average than rare variants (*R*_*cis*_ = −0.49, *R_trans_* = −0.58, P_cis_ < 2.2×10^− 16^, P_trans_ < 2.2×10^− 16^, Fig. 2C), consistent with previous results^13,16^. We also confirmed previous evidence^13,16^ that *cis* pQTL variants tend to have lower effect sizes as they get farther from the protein coding gene (Fig. 2D; Supp. Figure 5).

We and others have reported limited overlap of pQTL associations across tissues and with other molecular QTL^12,16^. We analyzed the overlap of the CSF pQTL with plasma and brain using two previously-published studies: first, summary statistics from a plasma pQTL analysis of over 35,000 individuals and approximately 5,000 proteins (SOMAscan5k; Supp. Table S11)^11^. In total, 4,735 aptamers overlapped between our meta-analysis and the large plasma pQTL study, covering 1,682 (72.6%, out of 2,316) of our CSF pQTL associations. Of the 1,682 possible shared signals with the plasma study^11^ (N = 35,559), 1,131 (67.2%, PP.H4 < 0.8) did not colocalize, representing novel and CSF-specific pQTLs (Fig. 2F). We identified a significantly higher ratio of CSF-specific *trans* associations compared to *cis* (548/734 *trans* vs 582/948 *cis*, P = 4.3×10^− 9^), consistent with previous work^16^. We also identified 634 additional novel index pQTL (27.4% of the 2,316 total CSF associations, 299 cis and 335 trans) that correspond to proteins that were not analyzed in the plasma study. A total of 1,765 CSF pQTL were not observed in the large plasma study.

Second, we compared to our previous brain (N = 380) and plasma (N = 529) pQTL (SOMAscan1.3k platform; Supp. Tables S12 & S13)^16^. For the brain dataset, 1,002 aptamers overlapped, corresponding to 465 associations. For the in-house plasma dataset, 862 aptamers overlapped, corresponding to 389 associations. We performed Bayesian colocalization for each pQTL association that had the corresponding aptamer present in one of the three datasets. We observed shared associations (posterior probability of one functional variant, PP.H4 ≥ 0.8) for just 32.8% (551/1,682) of the CSF pQTL with the larger plasma dataset^11^, 16.5% (64/389) with our previous plasma study, and 7.1% (33/465) with our previous brain study (Figs. 2F&G). The low overlap with brain may be partially driven by lower power to detect associations in the brain study (N = 380). In total we identified 1,720 novel index pQTL (74.3% of all CSF pQTL, 848 cis and 872 trans) that were either only significant in CSF (CSF-specific, 1,153) or were associated with proteins only measured in our analysis (true novel, 567). When analyzing all conditionally independent associations 2,448 were novel to CSF, of which 1,652 were considered CSF-specific and 796 were truly novel.

We next compared our *cis*-pQTL associations to multiple eQTL datasets from whole blood (GTEx^4^, N = 670; eQTLGen^5^, N = 31,684), neurologically-relevant tissues (GTEx cortex^4^, N = 205; MetaBrain^6^: cortex, N = 2,970; cerebellum, N = 492; basal ganglia, N = 208; hippocampus, N = 168; spinal cord, N = 108; and microglia^29^, N = 90). Of the 1,247 *cis*-pQTL associations, 29.2% colocalized (PP.H4 ≥ 0.8) with MetaBrain cortex eQTL, 22.4% with MetaBrain cerebellum, 16.6% with GTEx whole blood, 14.1% with eQTLGen whole blood, 9.5% with GTEx cortex, 7.5% with MetaBrain basal ganglia, 6.3% with MetaBrain hippocampus, 5.1% with MetaBrain spinal cord, and 4.7% with microglia (Fig. 2G, Supp. Tables S14-S22). We observed the largest number of shared associations with cortex and cerebellum even with relatively small sample sizes. In total, 567 (45.5%) *cis* CSF pQTL associations do not colocalize with any eQTL. Of the 680 *cis* signals that overlap between eQTL and pQTL, 83.1% (565) colocalize with at least one neurologically relevant tissue and 55.9% (380) are unique to these neurological tissues. Only 16.9% (115) are uniquely shared between CSF and whole blood. In total, 391 (31.4%) of our CSF *cis*-pQTL associations do not colocalize with any pQTL or eQTL dataset analyzed.

Consistent with previous research^16^, we observed a high degree of both tissue and molecule specificity for our CSF pQTL, with over 50% of the associations (*cis* and *trans*) not overlapping with any other dataset or tissue. When analyzing *cis* associations specifically, we observed the highest overlap with plasma pQTLs (366/1,247, 29.4% shared, Fig. 2G, Supp. Table S23), suggesting that regulation of proteins across tissues is more likely to be shared than that of eQTL vs pQTL even in the same tissues. This supports the notion that proteins have additional regulatory mechanisms beyond gene expression. When comparing our results to eQTL, we observed the highest overlap with the cortex and cerebellum (364 and 279 shared associations, 29.2% and 22.4%, Fig. 2G, Supp. Table S23), indicating CSF is a better proxy to brain than blood. These results support the fact that proteins are regulated at different levels compared to RNA that could include post-translational mechanisms (phosphorylation, glycosylation, cleavage, binding to receptors among others). These processes can contribute to localization of proteins, level of excretion from cells, and changes in protein conformation, all factors that can affect levels in the CSF.

## Pleiotropic regions regulate neurologically relevant pathways

Pleiotropic regions of the genome may be vital to regulation of multiple factors of biologically relevant pathways, but are often removed or not investigated in pQTL studies. To identify genomic regions that regulated multiple proteins, we grouped the index pQTL variants from each pQTL association through linkage disequilibrium, using a threshold of *R^2^* ≥ 0.1. We identified 194 regions that regulated levels of at least two proteins (271 regulating at least two aptamers, Fig. 2B, top; Supp. Figure 5; Supp. Table S24). Of these, 27 regions were associated with at least five proteins (37 with at least five aptamers), seven regions regulated at least ten, and three regions regulated more than 50.

Here, we prioritized the three most pleiotropic regions (chr3q28, chr6p22.2-21.32, and chr19q13.32; other regions are discussed in the extended results). Due to the complexity of the *HLA* region on chr6p22.2-21.32, we grouped all variants located in this locus together regardless of linkage disequilibrium information (see Methods). For these three regions, we first determined the genomic localization of the genes encoding each regulated protein. We then performed pathway and cell-type enrichment analysis to identify the cellular context of the regulated proteins. Finally, we performed a pheWAS using the GWAS Catalog^30^ to determine other traits regulated by the index pQTL variants in each region.

**Figure 3:**
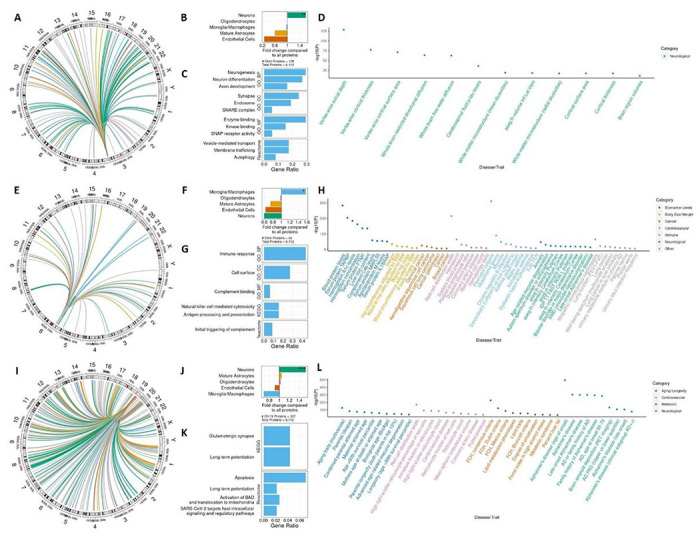
Three pleiotropic regions make up hotspots of protein regulation involved in neurological processes. **A.** Circos plot showing the genomic locations of all protein-coding genes whose proteins are regulated by the chr3q28 pleiotropic region. Colors represent the predominant brain-relevant cell type corresponding to that protein. **B.** Enrichment of proteins associated with the chr3 region in brain-relevant cell types (based on classification shown in **A**). Fold change was calculated based on the number of cell type-specific proteins in the region compared to the number in the entire SOMAscan7k panel. Enrichment p-value was calculated using a one-sided hypergeometric test. **C.** Selected pathways enriched for proteins associated with the chr3 region. Gene Ratio represents the proportion of all proteins associated with the region that are part of each pathway. **D.** PheWAS of index pQTL SNPs located in the chr3 region, as found in the GWAS catalog^30^. **E.** Circos plot showing the genomic locations of all protein-coding genes whose proteins are associated with the chr6p22.2-21.32 pleiotropic region. **F.** Enrichment of proteins associated with the chr6 region in brain-relevant cell types. **G.** Selected pathways enriched for proteins associated with the chr6 region. **H.** Selected traits and diseases associated with index pQTL SNPs located in the chr6 region, as determined by the GWAS catalog. **I.** Circos plot showing the genomic locations of all protein-coding genes whose proteins are regulated by the chr19q13.32 pleiotropic region. **J.** Enrichment of proteins associated with the chr19 pleiotropic region in brain-relevant cell types. **K.** Selected pathways enriched for proteins associated with the chr19 region. **L.** Selected traits and diseases associated with index pQTL SNPs located in the chr19 region, as determined by the GWAS catalog.

The chr3q28 pleiotropic region is intergenic (located between *GMNC* and *OSTN*) and consists of eleven index variants corresponding to one LD block (*R^2^* > 0.5, Supp. Figure 20F; two blocks when using *R^2^* = 0.8, Supp. Figure 10G) associated with 130 unique proteins (136 aptamers; Fig. 3A, Supp. Table S24), all of them *trans*. No pQTLs was observed in this region in plasma^11^, suggesting this is a CSF-specific pQTL hotspot. Proteins regulated by this region included five members of the syntaxin family (STX2, 3, 7, 8, and 12) involved in synapse function^31^ and five ephrin family members (EPHA7, EFNB1-3, EPHB2) involved in neural development and memory^32,33^. For each of the proteins regulated in the region, we analyzed brain-relevant cell-type expression data^34^ (Supp. Table S25) to determine cell specificity and observed a significant enrichment of neuron-specificity (P = 0.001, FC = 1.588, Fig. 3B, Supp. Figure 11, Supp. Table S26), suggesting functional relevance of this region for brain-related traits. In addition, we observed 234 enriched pathways associated with the 130 proteins. The 26 most-significant pathways were dedicated exclusively to neuronal and cell surface pathways (Fig. 3C, Supp. Figure 10, Supp. Table S27). These included neuron projection development (GO: 0031175, 32/123 analyzed proteins, P = 3.99×10^− 9^), cell junction (GO: 0030054, 44/127, P = 1.22×10^− 8^), axon development (GO: 0061564, 20/123, P = 4.96×10^− 7^), and SNAP receptor activity (GO: 0048812, 7/126, P = 5.93×10^− 7^). A PheWAS of the index variants in this region identified twelve traits associated with index pQTL variants, eleven of which were measures of brain morphology, volume, or surface area (Fig. 3D, Supp. Table S28). These include highly significant associations for vertex-wise sulcal depth^35^ (P = 1×10^− 128^), white matter mean diffusivity^36^ (P = 1×10^− 19^), and cortical surface area^37^ (P = 1×10^− 17^). Importantly, we also observed an association between rs9877502 and CSF levels of phosphorylated tau (P = 1×10^− 36^), a pathological hallmark for AD^38^ and a key biomarker. Additionally, *OSTN*, flanking the 3’ end of this region, is highly expressed in human neurons^39^ and regulates dendritic growth in the human brain^40^, suggesting regulation of *OSTN* may be driving the associations in the region. The highly neuronal-specific nature of the associated proteins, the link between *OSTN* and dendritic growth, and the numerous associations with brain morphology in this region highlight its importance for neurological development.

The chr6p22.2-21.32 pleiotropic region spanned about 8MB in the *HLA* region and was flanked by *HIST1H2AA* to *RPL12P1* based on literature-defined boundaries^41^. This region corresponds to 156 associations for 140 index pQTL variants in 65 LD blocks (*R^2^* > 0.5) associated with 68 unique proteins (74 aptamers; Fig. 3E, Supp. Table S24). The same region in plasma contained 1,757 associations^11^, significantly more than CSF (9.72% of all plasma associations vs 6.74% of all CSF associations; P = 4.35×10^− 6^). In CSF, we identified 32 *cis* associations for 28 aptamers and 23 unique proteins and 124 *trans* associations for 60 aptamers and 56 unique proteins. These included multiple complement components such as complement C2 (top index pQTL SNP P = 8.32×10^− 32^), complement factor B (top index pQTL SNP P = 1.86×10^− 31^), and C4a anaphylatoxin (top index pQTL SNP P = 1.86×10^− 21^). The proteins with pQTL in this region were enriched in microglia and macrophages (P = 0.03, FC = 1.574, Fig. 3F, Supp. Figure 11, Supp. Table S26). Pathway analysis identified 64 total enriched pathways among proteins regulated by variants in this region (Fig. 3G, Supp. Figure 12, Supp. Table S29). These were highly immune-specific, including regulation of the immune response (GO: 0050776, 23/64 analyzed proteins, P = 2.98×10^− 10^), leukocyte mediated immunity (GO: 0002443, 14/64, P = 1.38×10^− 7^), and antigen processing and presentation (KEGG: hsa04612, 6/39, P = 7.03×10^− 6^). Through a pheWAS of the index pQTL variants in this region, we identified 160 associations with traits and diseases (Fig. 3H, Supp. Table S30). These included autoimmune disorders like asthma^42^ (P = 8×10^− 14^) and myositis^43^ (P = 3×10^− 49^) and neurodegenerative disorders including Parkinson’s disease^44^ (P = 4×10^− 17^) and AD^45^ (P = 3×10^− 14^). The shared associations in this region with both immunological and neurodegenerative traits adds evidence to the known relationship between these two processes^46^.

In CSF, the region associated with the largest number of proteins corresponds to eleven index pQTL variants in three LD blocks (*R^2^* > 0.5, Supp. Figure 13D; seven LD blocks with *R^2^* = 0.8; Supp. Figure 13E) on chr19q13.32 in the *APOE* gene region. Variants in this region are associated with the levels of 331 proteins (337 aptamers; Fig. 3I, Supp. Table S24), of which three (two isoforms of APOE and APOC2) are in *cis*. This locus is known to regulate the levels of many proteins in plasma and CSF^11,12,16^. We observed significantly more associations located in this region in CSF than in plasma^11^ (14.6% of all CSF pQTL vs 0.67% of all plasma pQTL, P < 2.2×10^− 16^). *APOE* is the strongest genetic risk factor for AD^47^ and has been associated with at least eighteen other diseases, including heart disease and high cholesterol^48^. We observe associations in this region for known AD biomarkers, including four members of the 14-3-3 protein family (YWHAB, YWHAE, YWHAG, and YWHAZ), and neurofilament light and heavy chains (NEFL and NEFH)^49,50^. Calcineurin (PPP3R1), associated with both phosphorylated tau levels and rate of decline in AD^51^, is also genetically regulated by this region. Interestingly, although we observed an enrichment in neurons of proteins regulated by variants in this region (P = 7.93×10^− 6^, FC = 1.53, Fig. 3J, Supp. Figure 11, Supp. Table S26), APOE itself was specific to astrocytes (72.4% of expression in astrocytes, Supp. Table S25). This suggests a potential cell-to-cell communication between astrocytes and neurons. Pathway analysis results support this hypothesis (Fig. 3K, Supp. Figure 13), as we observe enrichment for seventeen pathways including glutamatergic synapse (KEGG: hsa04724, P = 5.1×10^− 4^), assembly and cell surface presentation of NMDA receptors (Reactome: R-HSA-9609736, P = 1.66×10^− 4^), and axon development (GO: 0061564, P = 6.58×10^− 5^). We also observe pathways enriched for apoptotic function, suggesting a potential role of APOE^52–54^ in cell death. Alternatively, this finding could be tagging neuronal death and neuronal protein release, which would be expected driven to individuals with neurological disease, as and be associated with *APOE4*. In order to determine if this is the case, we analyzed these variants in individuals that are cognitively normal and biomarker negative (AT^−^) and compared them to those who are biomarker positive (AT^+^). We found that for these 337 aptamer-SNPs pairs, 299 showed no significant difference in effect size between the AT^−^ and AT^+^ analyses, suggesting that this finding is not just an artifact of disease status. APOE variants regulate the levels of these proteins in both healthy and diseases individuals. Pathways related with the apoptotic function include activation of BH3-only proteins (Reactome: R-HSA-114452, P = 5.67×10^− 5^) and apoptosis itself (Reactome: R-HSA-109581, P = 2.4×10^− 4^, Supp. Table S31). Our PheWAS of the three independent signals in this locus identified 1,232 associated traits, highlighting the highly pleiotropic nature of this region and its involvement in several diseases and risk factors. These traits were largely involved in aging, cardiovascular, metabolic, and neurological traits, including lifespan, coronary artery disease, familial combined hyperlipidemia, and AD (Fig. 3L, Supp. Table S32).

Overall, we observed three highly pleiotropic regions that regulated the levels of many CSF proteins located on chromosomes 3q28, 6p22.2-21.32, and 19q13.32. The chromosome 6 and 19 regions, centered around the *HLA* and *APOE* loci, respectively, were also observed in plasma, although our analyses indicate that the *APOE* region show a higher degree of pleiotropy in CSF than plasma^11^ (P < 2.2×10^− 16^) and the opposite for chr 6 (P = 4.35×10^− 6^). In CSF both the chr3q28 and 19q13.32 regions were enriched for neuronal proteins and traits and were more pleiotropic in CSF than plasma, suggesting even pQTL hotspots show differences across tissues. While these regions are often overlooked in QTL analyses, we highlight that they represent master regulatory regions that are vital to the understanding of disease and important biological processes, and that to fully understand protein-protein interactions and proteins that are part of the same pathway it is instrumental to further explore these pleiotropic regions.

## Novel proteins associated with Alzheimer’s disease

Approaches integrating QTL with disease GWAS have been successful at resolving GWAS loci to prioritize causal genes. We sought to build upon this in the context of AD by utilizing three independent and complementary approaches: estimation and correlation of protein levels in AD using shared genetic etiology through proteome-wide association study (PWAS)^55,56^, prioritization of putative causal proteins for AD through Mendelian randomization (MR)^57^, and identification of shared genetic variants associated with both protein level and AD through colocalization (COLOC)^58,59^.

We performed a PWAS using the FUSION framework^55^. We first calculated variant weights for each protein and for each association separately for those aptamers with at least one study-wide pQTL. We then obtained the genetically estimated protein levels based on these weights and correlated them with disease status using AD GWAS summary statistics (Supp. Figure 14). Pathway enrichment analysis results and directionality for all PWAS-significant aptamers are available in Supp. Figures 14&15 and Supp. Table S33&S34. Because all proteins with associations in the *APOE* region were associated with AD risk (Supp. Figure 16), we removed *APOF*-associated proteins and recalculated pathway enrichment (Supp. Figure 17, Supp. Table S35). Finally, all proteins associated with AD through a *cis* locus are detailed in Supp. Figure 18 and through a *trans* locus (besides the *APOE* and *HLA* regions) in Supp. Figure 19. After excluding pleiotropic regions (those associated with five or more aptamers), 115 associations for 107 aptamers and 97 proteins were significant in the PWAS analyses, including 81 that were driven by *cis*-pQTL and 34 by *trans* (Supp. Table S33).

**Figure 4:**
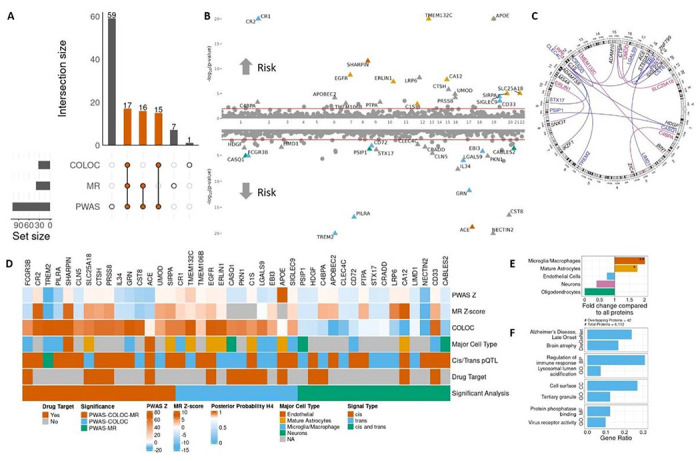
AD-related proteins are enriched in microglia and immune-relevant pathways. Figure 4. **A.** Upset plot detailing the protein overlap between PWAS, Colocalization, and Mendelian Randomization (MR) after removal of all associations in pleiotropic regions (N ≥ 5). **B.** Miami plot detailing the proteins that are significant in at least two of PWAS, COLOC, and MR. X-axis: chromosome position of the transcription start site of the protein coding gene; y-axis: PWAS −log10(P) for association with AD. Red line represents B&H FDR-corrected p-value threshold. Top plot: Positive association with AD; Bottom plot: negative association with AD. Triangle-shaped points correspond to the 42 proteins (48 aptamers) prioritized in (**A**). Color represents the predominant brain-relevant cell type for that protein. **C.** Circos plot showing *trans* associations linked to AD through two or more methods. Proteins labeled in red or blue are associated with AD through a transassociation (red, positively associated; blue, negatively associated). Links start at the TSS of the associated protein-coding gene and end at the index pQTL variant. Associations are labeled in black by the nearest gene to the pQTL. **D.** Details of the 42 proteins that overlap between at least two of PWAS, colocalization, and MR. PWAS Z & MR Z: orange represents positive Z-score, blue represents negative z-score. COLOC: color represents posterior probability of sharing a genetic signal between pQTL and AD (Orange: PP.H4 > 0.8; blue: PP.H4 < 0.8). Major cell type: Predominant brain-relevant cell type for proteins of interest. Drug Target: Proteins targeted by a molecule as described in the DrugBank database. Significant Analysis: Of PWAS, COLOC, and MR, orange corresponds to reaching inclusion threshold in all three; blue corresponds to reaching inclusion threshold in only PWAS & COLOC, green corresponds to reaching inclusion threshold in only PWAS and MR. **E.** Enrichment of 42 proteins from (**D**) in brain-relevant cell types. **: P < 0.01; *: P < 0.05. **F.** Selected gene sets enriched for proteins from (**D**). Gene Ratio: proportion of the 42 proteins/genes that are part of the pathway.

We next performed MR^57^ to identify proteins causal for AD, using the pQTL summary statistics and the Bellenguez et al. AD GWAS summary statistics^22^. For each aptamer, we selected significant *cis* and *trans* instrument variables through LD pruning on all variants with P < 5×10^− 8^, after removing pleiotropic regions. Our analyses identified 37 proteins (40 aptamers) as putative causal for AD (Fig. 4A, Supp. Table S36&S37) based on MR analyses (FDR-corrected P < 0.05) with confirmed directionality based on the Steiger test^60^ (Supp. Table S38). In addition, 33 of these 40 MR-significant aptamers were also FDR-significant in the PWAS analysis (Fig. 4A). *Cis*-specific MR for those with applicable variants confirmed all but one association (APOBEC2, Supp. Figure 21).

Finally, we performed COLOC^58,59^ between each of our 2,316 significant pQTL associations (*cis* and *trans*) and the AD GWAS. After exclusion of pleiotropic regions, we identified 28 proteins (33 aptamers) that colocalized with AD risk (Fig. 4A, Supp. Tables S39 & S40). After filtering of the colocalization results based on pleiotropy, 32 aptamers overlapped between PWAS and COLOC.

Of all proteins prioritized by PWAS, COLOC and MR, 15 (17 aptamers, eleven in *cis*, three in *trans*, and one in both *cis* and *trans*) were shared between all three of them and 42 proteins (48 aptamers, 31 *cis*, 17 *trans*) were significant between at least two of the methods (Figs. 4A–D; Supp. Table S41). Through cell-type enrichment analysis, we found that these 42 proteins were overrepresented in microglia/macrophages (N = 10, P = 0.008, FC = 2.01) and mature astrocytes (N = 8, P = 0.03, FC = 1.76, Fig. 4E, Supp. Figure 11, Supp. Table S26). We also performed pathway and gene set enrichment analyses and observed overrepresentation of the proteins in pathways relating to neurodegenerative traits, including late onset AD (DisGeNet:C0494463; P = 1.32×10^− 6^) and brain atrophy (DisGeNet:C4551584; P = 4.55×10^− 6^). Consistent with the cell type enrichment, these proteins were also present in immune pathways, including regulation of the immune response (GO:0050776; P = 1.58×10^− 5^) and virus receptor activity (GO:0001618; P = 6.46×10^− 4^, Fig. 4F, Supp. Figure 20, Supp. Table S42).

## Proteins supported by multiple methods

These (n = 42) proteins associated with AD based on our analyses (PWAS, COLOC and/or MR) 1) provide additional evidence of functionality for some of the nominated AD gene loci, 2) nominate alternative genes in those loci, and 3) identify novel genes implicated in AD that have not been reported before.

Our findings support the nominated functional gene for ten of the GWAS-identified^22^ loci: *APOE, ACE, CR1, CTSH, EGFR, GRN, IL34, SHARPIN, TMEM106B*, and *TREM2*. These include putatively protective effects (as identified through PWAS and MR) for ACE in *cis* at 17q23.3 (PWAS Z=−9.04, MR β=−0.206), TREM2 in *cis* at 6p21.1 (PWAS Z=−14.5, MR β=−0.236), TREM2 in *trans* at 11q12.2 (*MS4A* locus, PWAS Z=−10.03, MR β=−0.236), and GRN in *cis* at 17q21.31 (PWAS Z=−7.05, MR β=−0.22). We also identified proposed proteins associated with increased risk, including CTSH in *cis* at 15q25.1 (PWAS Z = 4.81, MR β = 0.041) and SHARPIN in *cis* at 8q24.3 (PWAS Z = 6.98, MR β = 0.246). These results offer more evidence for established candidate genes like *TREM2* and *CR1*, while also reaffirming the genes prioritized at newly-identified loci (*SHARPIN, EGFR, CTSH*, among others).

We identified alternative candidate proteins at four AD risk loci. First, the association at 1q32.2 has been associated with *CR1* for years. While we observed an association between CR1 and AD through PWAS and COLOC, we also observed the same positive association with CR2 protein (index pQTL variant rs679515) in both PWAS and MR (PWAS Z = 11.85, PWAS P = 1.94×10^− 32^, MR P = 3.39×10^− 13^, COLOC PP.H4 = 0.996, Supp. Figure 22). Most studies have nominated *CR1* as the functional AD gene in this region^61–63^, but our analyses also nominate *CR2* as a potential additional factor affecting this locus. The similarities in structure and function between CR1 and CR2^64^ make it plausible that both may play a role in neuroinflammation known to be implicated in AD pathogenesis^65^. At the 7q22.1 AD locus, the proposed genes were *ZCWPW1* and *NYAP1*. However, the causal gene has been unclear, with *PILRA* and *PILRB* also nominated^66^. Our analyses indicate that *PILRA* can be also a functional gene in this locus. *Cis* levels of PILRA (index pQTL variant rs1859788) were negatively associated with AD (PWAS Z = −8.532, PWAS P = 1.44×10^− 17^, MR P = 4.06×10^− 11^, PP.H4 = 0.995, Supp. Figure 22). This supports previous research suggesting altered ligand binding with PILRA offers a protective effect on AD^67^, with the index pQTL variant identified being described as the likely causal allele. The index variant has also been fine-mapped as the likely causal variant at the locus in another GWAS study^68^. Additionally, at the 16p11.2 AD locus, we nominate *PRSS8* as the candidate gene instead of *KAT8*. *cis*-regulated levels of PRSS8 (index pQTL variant rs1978485) were associated with increasing risk of AD (PWAS Z = 4.01, PWAS P = 5.95×10^− 5^, MR P = 2.43×10^− 6^). Using COLOC-SuSiE^59^, we identified three credible sets of causal variants affecting PRSS8 levels, with only one colocalizing with AD risk (PP.H4 = 0.977, Supp. Figure 22), highlighting the complexity involved in GWAS locus resolution. This protein was also identified as being associated with AD in plasma^12^, albeit with an opposite direction of effect. This directionality difference between tissues has been reported for other proteins for AD in previous analyses^10^. Finally, at 19q13.32, we identify *NECTIN2* (index pQTL variant rs3810143) as an additional candidate gene near *APOE*. CSF NECTIN2 levels were associated with lower risk of AD (PWAS Z = −12, MR β = −0.685). The protein serves as a receptor for multiple Herpes Simplex Virus (HSV) forms^69^, which have repeatedly been linked to AD^70^. Recent work from our group has also identified an association in the *NECTIN2* region for CSF TREM2 levels (in press), supporting its relevance in AD biology. Through integration of AD GWAS with CSF pQTL, we identify proteins that differ from those prioritized previously and may constitute new targets.

While the power of AD GWAS studies is constantly increasing, undiscovered associations that may be found by including larger samples size still exist. In support of this, we also identified twelve proteins (APOBEC2, C1S, CA12, CABLES2, CD33, CD72, CLN5, FCGR3B, HDGF, PTPA, SIGLEC9, and SIRPA, all cis) whose associated loci do not reach genome-wide significance in GWAS studies, but are associated with AD in our analyses. Of these proteins, three were supported by all three analyses (PWAS, COLOC, and MR): FCGR3B, CLN5 and SIRPA. On chromosome 1, higher genetically estimated CSF levels of FCGR3B (*cis* association, index variant rs4379692) were associated with lower risk of AD (PWAS Z=−4.45, P = 8.42×10^− 6^, MR β=−0.0195, P = 9.64×10^− 8^) at a locus that reaches suggestive significance for AD (PP.H4 = 0.937).

Low *FCGR3B* copy number has been associated with autoimmune disease^71^, supporting an important role in immune function also associated with AD. We observed a negative association between *cis*-regulated levels of CLN5 and AD (chr13:77000848:A:G, PWAS Z=−4.21, PWAS P = 2.59×10^− 5^, MR P = 3.29×10^− 5^, PP.H4 = 0.946, Supp. Fig. S21). Variants in *CLN5* cause a form of neuronal ceroid lipofuscinosis, a lysosomal storage disorder^72^. CLN5 defects have also been linked to retromer dysfunction, which has previously been identified in AD^73,74^. On chromosome 20, *cis*-regulated levels of SIRPA were positively associated with AD risk (rs17855616, PWAS Z = 4.41, PWAS P = 1.02×10^− 5^, MR P = 1.37×10^− 5^, PP.H4 = 0.959). Loss of SIRPA in microglia has been associated with increased synapse loss^75^ and its binding partner, CD47, is upregulated in synapses in AD and may act through shielding of synapses from microglia pruning^76^. These proteins highlight the need for continued growth of AD GWAS to larger sample sizes. For discussion of the other proteins, see the Extended Results.

In addition, we also identified 17 proteins contributing to AD through at least two of PWAS, MR, and/or COLOC whose associations are driven by *trans*-pQTL including TMEM132C, LRP6, CRADD, and STX17 among others (Table S41, Fig. 4C). These findings suggest novel protein-protein interactions that have not been identified before, similar to the case of TREM2 levels regulated by *trans*-pQTL in the *MS4A* locus^7^ that we have replicated here. First, the *BIN1* locus is one of the regions with the strongest association with AD^22^. The causal link between *BIN1* and AD is unclear, but it has been connected to increased risk through interaction with tau^77^. We found that levels of TMEM132C were associated with the *BIN1* locus that colocalized with the AD risk signal (PP.H4 = 0.999, Supp. Table S39). CSF levels of TMEM132C regulated by this locus were associated with increased risk of AD (PWAS Z = 20.1, PP.H4 = 0.999, Supp. Fig. S21). While little is known about *TMEM132C* specifically, its family members have been linked to panic disorders^78^ and insomnia^79^, suggesting an important brain function potentially through interaction with the actin cytoskeleton^80^. This protein also has a pQTL at the *APOE* locus discussed above. We also observed *trans*-pQTL for both STX17 and CRADD at 11q24.3 near *ADAMTS8* (index pQTL variant rs3740888). Mutations in *CRADD* cause intellectual disability^81^ and the protein contributes to apoptosis of neurons^82^, potentially mediating a protective effect on AD through increased apoptosis of diseased neurons. Like TMEM132C, CRADD is also associated with the *APOE* locus discussed above. STX17 is a member of the SNARE complex and is vital for the fusion of autophagosomes with lysosomes^83^ and interruption of this function is associated with axonal dystrophy, a feature of AD^84^. Both proteins were associated with decreased risk of AD at this locus (STX17 PWAS Z=−3.84 MR β=−0.062; CRADD PWAS Z=−3.53, MR β=−0.102). These analyses suggest that ADAMTS8, STX17 and CRADD are part of the same pathway, potentially contributing to disease through autophagosomal-lysosomal pathways. Additionally, we observed an association between CSF levels of LRP6 and intergenic variants in *ADAM10* at 15q21.3 (index pQTL variant rs1427281). ADAM10 contributes to cleavage of amyloid precursor protein into neuroprotective components^85^ and overexpression of it in a mouse model of AD was found to reduce amyloid plaque burden^86^ and lessen memory deficits^87^. Levels of LRP6 regulated by this locus were associated with increased risk of AD (PWAS Z = 5.81, MR β = 0.189). LRP6 has been found to function in Wnt signaling vital to synapse function and variants in *LRP6* have been associated with synapse degeneration in AD^88^. Variants at this locus may contribute to AD by increasing secretion of LRP6 to CSF and thereby decreasing the cellular levels of the protein. The integration of *trans*-pQTL with AD performed here identifies novel protein-protein interactions that help to explain the disrupted pathways observed in AD. All other proteins prioritized through *trans* associations are discussed in the Extended Results.

Consistent with our cell-type results that showed enrichment of our 42 proteins in microglia/macrophages (N = 10, P = 0.008, FC = 2.01) and mature astrocytes (N = 8, P = 0.03, FC = 1.76, Fig. 4D, Supp. Figure 11, Supp. Table S26), these AD-associated proteins were enriched in immune function (GO:Regulation of immune response, P = 1.58×10^− 5^, Fig. 4E, Supp. Table S42). Immune-relevant proteins include TREM2 (*cis* association at 6p21.1 and *trans* at 11q12.2), which is well-known for its role in microglia’s response to neurodegeneration^89^. It is targeted for processing to the soluble form by ADAM10^90^, whose genetic locus was found to also regulate LRP6 levels above. TREM2 complexes with DAP12, which regulates activation of microglia through its immunoreceptor tyrosine-based activation motif (ITAM)^91^. IL34, also identified here, interacts with CSF1R on the surface of microglia leading to microglial activation^92^. Several of our identified proteins, including PILRA^93^, CD33^94^, and SIGLEC9^95^, also contain ITAM or immunoreceptor tyrosine inhibitory motif (ITIM) domains. ITAM-containing proteins in coordination with ITIM-containing proteins regulate processes including microglial phagocytosis and apoptosis^96^. We also identified other proteins involved in immune response. Variants in *SHARPIN* were associated with AD through reduction of the NF-κB-mediated immune response^97^. C1S and C4BPA are both involved in the complement system^98,99^.

Another relevant pathway for these proteins involves lysosomal dysfunction which is known to be implicated in AD pathogenesis^100^. Our analyses not only support this process but also extend its ties to AD by adding novel causal proteins linked to the lysosome (GO:Lysosomal lumen acidification including CLN5, GRN, TMEM106B, P = 1.23×10^− 5^, Fig. 4E, Supp. Table S42). As stated above, mutations in *CLN5* contribute to a form of neuronal ceroid lipofuscinosis^72^. Neurons deficient in CLN5 protein showed impaired lysosomal activity and movement, suggesting the vitality of CLN5 to lysosome function^72^. In AD, autophagosomes showed an inability to fuse with lysosomes, causing accumulation in neurites^101^. In addition, STX17, associated in *trans* near *ADAMTS8*, is vital to the normal function of this process and depletion of STX17 leads to autophagosome accumulation^83,102^. We also identified an increased risk for AD associated with CTSH (Fig. 4B, Supp. Table S33). This is consistent with a previous work, wherein increased mRNA levels of CTSH were observed in AD patients and microglia with *CTSH* knockouts showed increased Aβ phagocytosis^103^. Cathepsins are known to function in proteolysis of lysosomal proteins^104^. Interestingly, CST8, a protein we associated with decreased risk of AD, is part of a protein family that inhibits the activity of cathepsins, offering a potential mechanism for its protective effect^105^. We also identified two proteins, GRN and TMEM106B, that are typically associated with frontotemporal dementia (FTD)^106,107^ but were also identified in the latest AD risk GWAS^22^. Both proteins are implicated in lysosomal function^108,109^. We prioritized two pathways through which AD pathology may develop. First, we identified numerous proteins involved in immune function, prioritizing those involved in ITAM/ITIM signaling. Second, we characterized potential functions of lysosomal proteins through interruption of autophagosome-lysosome fusion.

Finally, we searched DrugBank^110^ to identify potential therapeutic agents targeting the identified AD-associated proteins that may be repurposed. Of the 42 prioritized proteins, 15 had at least one reported molecule in DrugBank (Fig. 4C). FCGR3B is targeted by cetuximab (DrugBank accession number DB00002), a chemotherapy agent. Cetuximab has been associated with decreased risk of AD^111^, making it a potential drug-repurposing target (Supp. Table S5). Glutamic acid (DB00142) targets SLC25A18 and is heavily involved in excitatory signaling in neurons^112^. SLC25A18’s function as a mitochondrial glutamate transporter may be abnormal in AD^113^. CASQ1 is a ligand for calcium (DB11093), and studies have suggested that targeting calcium receptors may be beneficial in AD^114^. Finally, CA12 is targeted by carbonic anhydrase inhibitors like acetazolamide (DB00819), which was shown to reduce Aβ-induced mitochondrial toxicity^115^. All associated drugs can be seen in Supp. Table S5. Through our analyses, we identify numerous causal and druggable proteins for AD. These drugs, or others developed to target these proteins, may offer promising targets for AD treatment in the future.

## Identified proteins successfully predict AD biomarker status

**Figure 5:**
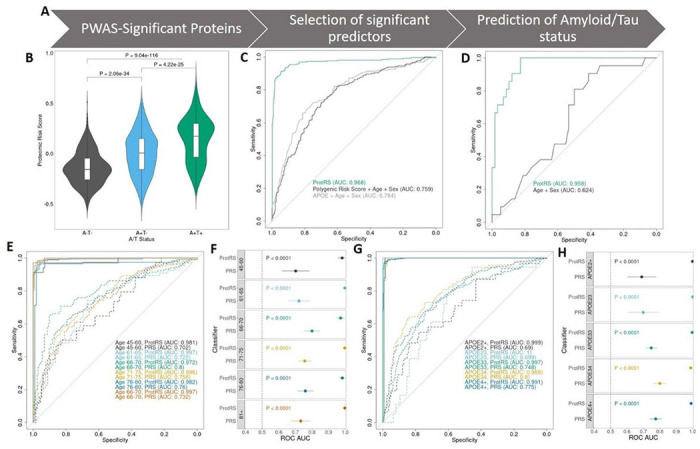
Protein-based prediction of amyloid/tau positivity is more accurate than a polygenic risk score. **A.** General schematic of proteomic risk score workflow. **B.** Violin plot of proteomic risk score values across amyloid/tau negative samples (gray, N = 889), amyloid positive/tau negative samples (blue, N = 496), and amyloid/tau positive samples (green, N = 775). Significance was calculated using the Wilcoxon rank-sum test. **C.** Receiver-operator characteristic (ROC) curves predicting amyloid/tau positivity vs negativity in an internal replication dataset. Polygenic risk score calculated including the *APOE* region using a p-value threshold of 5X10^− 8^. APOE status was based on APOE ε2, ε3, and ε4 carrier status (rs7412 and rs429358 genotype) for each allele. **D.** Replication of proteomic risk score in an external dataset (Stanford ADRC). **E.** Age-stratified ROC curves predicting amyloid/tau positivity vs negativity. Solid lines: LASSO-based proteomic risk score. Dashed lines: PRS + sex. **F.** Whisker plot of area under the curve and 90% confidence interval from ROC curves plotted in (**E**), grouped by age bracket. **G.** APOE-genotype stratified ROC curves. Solid lines: LASSO-based proteomic risk score. Dashed lines: PRS+ age + sex. Stratified groups are determined by APOE ε allele carrier status. **H.** Whisker plot of area under the curve and 90% confidence interval from ROC curves plotted in (**G**). Protein-based and genetic-based approaches are grouped by APOE genotype.

The identification of accurate tools to prioritize individuals at risk of AD is a vital focus for the field. We sought to compare the predictive power of the prioritized proteins to classify individuals who were positive for two established AD biomarkers: CSF amyloid beta-42 and CSF phosphorylated tau-181 protein. We developed a proteomic risk score based on the transcriptional risk score (TRS) approach^116^(Fig. 5A). Briefly, we started with the normalized aptamer levels (z-score) for all proteins associated with AD through PWAS (N = 456) in each individual. Using the TRS framework, we harmonized the aptamer levels so that higher proteomic risk score would always increase AD risk. The average per-individual PWAS protein z-score was significantly associated with amyloid/tau status (P for A−T− vs A + T + = 9.04×10^− 116^, Fig. 5B). We then split our dataset into training and testing datasets (training: ADNI, FACE; testing: MAP, Barcelona-1, consistent with our discovery and replication for the pQTL mapping). We used LASSO regression to select significant predictors (N = 162, aptamers with non-zero weights are denoted in Supp. Table S33) in the training dataset. We then used the calculated weights (designated ProtRS) to predict amyloid/tau (AT) positivity in the testing dataset, and additional independent dataset (Stanford Dataset), as well as in age-stratified and *APOE* genotype-stratified analyses.

The ProtRS showed highly accurate prediction of AT status in both the training (N_A−T−_ = 355, N_A+T+_ = 471, AUC = 1, Supp. Fig. S23) and in the testing datasets (N_A−T−_ = 429, N_A+T+_ = 261, AUC = 0.968, 90% CI = 0.955–0.981, Fig. 5C, Supp. Tables S44 & S45). In order to further validate this prediction model, we analyzed a third independent cohort (Stanford ADRC, N_A−T−_ = 58, N_A+T+_ = 21). These samples were quantified using the SOMAscan5k assay, so around half of the proteins in the model were missing (288/456 measured, 106/162 significant predictors measured). Even with potentially losing some effectiveness due to missing measurements, we still observed high predictive ability in the independent dataset (AUC = 0.958, CI = 0.920–0.996, Fig. 5D, Supp. Table S44).

Given the prioritized proteins were identified through genetics, we also compared the ProtRS to a model based on a polygenic risk score calculated using the AD risk GWAS^22^ (including the *APOE* region) with age and sex as well as a model combining *APOE* ε2 and ε4 genotype with age and sex. Both genetics-focused models showed AUC similar to other previous studies for PRS (AUC_PRS_ = 0.759, CI_PRS_ = 0.729–0.790; AUC_*APOE*_) = 0.784, CI_*APOE*_ = 0.748–0.820, Fig. 5C, Supp. Table S44) but the ProtRS showed significantly increased AUC compared to both models (ProtRS vs PRS P < 2.2×10^− 16^, ProtRS vs *APOE* P = 8.56×10^− 15^, Supp. Table S45). These results suggest a highly accurate and repeatable prediction model for AT status. We find that proteins prioritized through genetics significantly improve upon variant-based prediction models, supporting the use of genetics to prioritize potential disease biomarkers.

As protein levels change with time while genotype stays static, we wanted to determine if the ProtRS could predict AD risk at different ages. We stratified our samples by age bracket and compared our ProtRS to PRS (including sex; Fig. 5E, Supp. Tables S44 & S45). Our ProtRS model showed improved predictive ability compared to PRS for individuals in all age brackets (45–60, 61–65, 66–70, 71–75, 76–80, and 81+; P ≤ 5.80×10^− 9^, ΔAUC ≥ 17.2%, Fig. 5F). The ProtRS was especially accurate at predicting status in younger individuals compared to PRS, exhibiting an AUC of 0.981 in the 45–60 bracket (CI 0.955-1.00) compared to only 0.702 when using PRS (CI = 0.620–0.785, P = 5.80×10^− 9^, ΔAUC = 0.279). This highly significant improvement also persisted for individuals aged 61–65 (P = 7.54×10^− 12^, ΔAUC = 0.255).

PRSs for AD are heavily dependent on the inclusion of *APOE ε2* and *ε4* genotype, with knowledge of those genotypes alone being more predictive than a pruned model containing the other genome-wide variants^117^. We stratified our samples by *APOE* genotype to analyze the effectiveness of the ProtRS to predict AT status compared to PRS excluding the *APOE* region. The PRS model also included age and sex as covariates. The ProtRS outperformed the PRS with a significantly higher AUC across all genotype categories (ΔAUC ≥ 0.189, P ≤ 5.70×10^− 9^, Figs. 5G & 5H). While both methods performed well for individuals with the *APOE* ε4 allele, the PRS decreased in effectiveness in *APOE* ε33 or ε2 carriers while the ProtRS was consistent across genotypes. When predicting AT status in *ε2* carriers, the ProtRS had an AUC of 0.999 (CI 0.998-1.00) while the PRS showed an AUC of 0.690 (CI 0.603–0.778, P = 5.70×10^− 9^, ΔAUC = 0.309, Supp. Tables S44, S45). The ProtRS also had an AUC of 0.997 for predicting A/T status in *ε33* carriers (CI 0.995–0.999), significantly better than the PRS with an AUC of 0.0.748 (CI 0.714–0.782, P < 2.20×10^− 16^).

Across ages and *APOE* genotypes, the ProtRS developed based on AD-associated proteins is highly accurate and consistently performs better than PRS at predicting amyloid/tau positivity. This is consistent with previous results, where risk scores based on molecules predict disease more effectively than genetic variants alone^116,118^. Our results were consistent across training, testing, and external replication datasets, supporting our model as being highly replicable. We also showed high accuracy in all age brackets and *APOE* genotypes, while the PRS was less accurate in younger individuals and those without the *APOE* ε4 allele.

## Discussion

Neurological disorders and diseases constituted the cause of nine million deaths in 2019 alone^119^, representing the second leading cause of death after heart disease^120^. However, the prevalence of these disorders has not translated effectively to treatments. Dementia is one of the ten most common causes of death worldwide^121^. AD causes 60–80% of these cases^122^, does not have a cure, and disease-modifying treatments have limited efficacy^123^. Large-scale genomic studies have identified over 70 genetic loci associated with AD^22^, offering many new potential treatment targets and additional knowledge about AD. Here, we have analyzed the genetic regulators of CSF protein levels and integrated them with AD genetics to nominate new proteins involved in AD. One method to prioritize genes potentially useful for treatment is through integration of disease GWAS with molecular QTL, but the majority of these datasets target molecules or factors that do not directly affect disease. Proteins, due to their active participation in biological processes, often are the direct contributing factors in traits. However, there is limited knowledge of the genetic regulators of protein levels. The two largest datasets both analyzed over 10,000 individuals^11,12,15^, but both included fewer proteins than our dataset and analyzed plasma proteomics only. Transfer of molecules including proteins from blood into the brain and vice-versa is highly regulated by the blood-brain barrier^124^, potentially limiting the overlap between protein regulation in the brain and blood. In addition, even for proteins that originate in the central nervous system and are present in plasma, their levels may be far lower and regulated differently than in CSF^8,9^.

CSF is highly neurologically relevant as the fluid that surrounds the brain and spinal cord, and a large portion of CSF proteins are derived from the central nervous system^125^. The ability to extract CSF from living individuals and its shared protein biology with the brain makes it highly relevant for the study of neurological disorders and diseases. Here, we present the largest-to-date proteogenomic analysis of CSF. This dataset presents an expansion of previous CSF pQTL work^16–20^ by tripling previous sample sizes (N = 3,107) and increasing the number of analyzed proteins by over sixfold (N = 7,028). Overall, we identified 3,373 independent study-wide significant pQTL associations (1,247 *cis*, 1,069 *trans*) for 1,786 proteins, corresponding to 28.9% of all analyzed proteins. We further confirmed that these are mainly unique to CSF, supporting the analysis of tissue-relevant proteomic datasets to better understand complex traits. Through these analyses, we show strong differences in protein regulation between tissues, emphasizing the need for tissue-relevant approaches for understanding brain-related traits.

While integration of GWAS with QTL has elucidated functional genes at many disease-associated loci, many genetic variants have not been connected to genes. In AD, novel loci have been identified without obvious causal genes^22,68^. To attempt to clarify the functional genes at some of these loci, we integrated our pQTL with summary statistics for AD through a proteome-wide association study, Mendelian randomization, and Bayesian colocalization. We identified 42 proteins associated with AD through at least two of these methods. These were enriched for microglia and astrocyte-specific proteins, supporting previous evidence of a strong immunological component in AD^126–128^. The proteins were also enriched in immune-relevant pathways (through proteins like TREM2^7,129^, CD33, IL34, and others) and lysosomal function (GRN, TMEM106B, CLN5 among others). These candidate proteins provide support for candidate causal genes and identify potential new pathways relevant to AD. Two of these proteins (ACE, CTSH) were also identified in an AD PWAS using brain tissue, highlighting the relevance of CSF to brain biology^56^. We also identify potential drug repurposing candidates for AD, including cetuximab, acetazolamide, and calcium receptor-targeting drugs.

Using the transcriptional risk score framework^116^, we developed a risk score based on AD-associated proteins and applied it for predicting CSF amyloid-tau positivity, a proxy for AD diagnosis^25^. As the proteins were identified through integration with AD GWAS^22^, we compared the proteomic risk score to a PRS calculated from the same GWAS. We showed high accuracy in both internal and external replication datasets and improved upon PRS in all facets, including in age-specific and *APOF*-specific contexts, highlighting the proximity of proteins to disease compared to genetics.

We note several limitations in our study warranting further investigation. We included only non-Hispanic white individuals in our pQTL analysis, which may limit the number of associations identified and applicability of our results to other ancestries. Additionally, due to the nature of aptamers, mutations occurring in binding sites of the protein-targeting aptamers may lead to extreme differences in measured protein abundance due to binding that may not be true abundance differences. While we performed AD-specific analyses using the largest AD GWAS to date^22^, this involved proxy-AD cases from UK Biobank that were not clinically diagnosed, potentially clouding the results with other forms of dementia or with individuals that do not develop AD. In addition, the gene prioritization was limited to the approximately 6,100 proteins that were present in the SOMAscan7k panel^24^, leaving many AD-associated regions unexplored. In many instances, the protein encoded by the candidate gene proposed in the AD GWAS was not included in our analysis, preventing us query those genes. Our cell-type analyses were also derived from healthy individuals and were based on RNA levels, which are known to correlate poorly with proteins. Investigation of the levels of these proteins in a cell-type specific and disease-stratified manner is an important direction.

While we have successfully identified novel candidate proteins in AD, we foresee this dataset being a useful resource for the investigation of all neurological traits. Previous evidence suggests that numerous candidate proteins can be identified for many neurological traits, even with smaller sample sizes^13,16^, and this dataset has already been applied to identify associations between proteins regulated by the *LRRK2* genetic locus and Parkinson’s disease^130^. We intend these results to add to the growing knowledge of genetic regulation of protein levels, and as such, we have made all summary statistics publicly available for use at https://www.niagads.org/knight-adrc-collection.

## Materials and Methods

### Ethics declarations

The ethics committee of Washington University School of Medicine in St. Louis approved this study.

All participants provided informed consent for their data and specimens to be used for this study. The study was approved by the institutional review board of Washington University School of Medicine in St. Louis.

### Cohorts

This study analyzed 4,223 cerebrospinal fluid proteomics samples from six cohorts (Alzheimer’s Disease Neuroimaging Initiative [ADNI], Dominantly Inherited Alzheimer’s Network [DIAN], Knight-ADRC Memory and Aging Project [MAP], Ace Alzheimer Center Barcelona (FACE), Barcelona-1, and Parkinson’s Progression Markers Initiative [PPMI]).

#### Knight ADRC MAP

The Knight ADRC at Washington University School of Medicine (St. Louis, MO, USA) recruits and longitudinally assesses community-dwelling adults older than 45 years old via prospective studies of memory and aging since 1979. All studies were approved by the Human Research Protection Office at Washington University, and written informed consent was obtained from all participants. The Memory and Aging Project at the Knight ADRC (Knight ADRC-MAP) involves longitudinal collection of biofluids (plasma, CSF, fibroblast), annual clinical assessments, neuropsychological testing, and neuroimaging studies, as well as collection of autopsied brain samples. Eligible participants may be asymptomatic or have mild dementia at the time of enrollment. All participants are required to participate in core study procedures, including annual longitudinal clinical assessments, neuropsychological testing, neuroimaging, and biofluid biomarker studies. Annual assessments of the participants were performed by experienced clinicians using a semi-structured interview with knowledgeable collateral source and the symptomatic individual in accordance with the Uniform Data Set protocol of the National Alzheimer’s Coordinating Center^131^, as well as a detailed neurological examination. Participants comprise Non-Hispanic White individuals from North America (82.5%) and African-Americans (13.3%). Samples have been obtained from over 5,510 participants, including 2,426 AD cases, 148 FTD, 88 DLB, and 2,156 cognitively normal healthy individuals. Autopsy material are available for over 1,182 participants including 474 with fresh frozen parietal brain tissue (https://dss.niagads.org/datasets/ng00127/ ). Multi-tissue (brain, CSF, and plasma), multi-omics data (genetics, epigenomics, transcriptomics, proteomics, and metabolomics) have been generated for the purpose of identifying novel risk and protective variants for dementia, and potential drug targets. Participants from the Knight ADRC were included in this study if they were cognitively unimpaired with a global clinical dementia rating (CDR) score of 0 at enrollment. A clinical diagnosis of incident dementia is considered by study clinicians at the conclusion of each annual assessment, integrating results from the clinical assessment and bedside measures of cognitive function^132^. Dementia was diagnosed according to the National Institute of Neurological Disorders and Stroke criteria^133^ and National Institute on Aging-Alzheimer’s Association Work Group criteria for participants assessed after 2011^134^. Diagnosis of AD dementia was made in accordance with criteria developed by working groups from the National Institute of Aging and the Alzheimer’s Association^134^. Diagnosis of vascular dementia conformed to the NINDS-AIREN criteria^135^. More information is available at knightadrc.wustl.edu.

#### DIAN

Cerebrospinal fluid samples and genetic data were obtained from the Dominantly Inherited Alzheimer’s Network (DIAN). Led by the Washington University in St. Louis School of Medicine, DIAN utilizes a family-based long-term cohort study to investigate Autosomal Dominant Alzheimer’s Disease (ADAD). Tissues collected (blood, CSF) are analyzed to detect changes in carriers of mutations causal for ADAD. The samples and data utilized in this study are from the 15th datafreeze (DF15). For more information about DIAN, visit dian.wustl.edu.

#### ADNI

Cerebrospinal fluid samples and genetic data used in the preparation of this manuscript were obtained from the Alzheimer’s Disease Neuroimaging Initiative (ADNI) database (adni.loni.usc.edu). ADNI was launched in 2003 as a public-private partnership, led by Principal Investigator Michael W. Weiner, MD. The primary goal of ADNI has been to test whether serial magnetic resonance imaging (MRI), positron emission tomography (PET), other biological markers, and clinical and neuropsychological assessment can be combined to measure the progression of mild cognitive impairment (MCI) and early Alzheimer’s disease (AD).

#### Ace Alzheimer Center Barcelona

We obtained cerebrospinal fluid samples and genetic data from Ace Alzheimer Center Barcelona, a private non-profit group dedicated to the study of Alzheimer’s disease. Headquartered in Barcelona, Ace Alzheimer Center Barcelona was founded in 1995 and has diagnosed over 30,000 patients, collected 20,000 blood and 1,831 cerebrospinal fluid samples, analyzed almost 13,000 genetic samples^136,137^, and participated in almost 150 clinical trials during its existence. For more details, visit www.fundacioace.com/en.

#### Barcelona-1

Cerebrospinal fluid samples and genetic data were obtained from Barcelona-1, a study led by the University Hospital Mutua de Terrassa in Terrassa, Spain. Barcelona-1 is a longitudinal study consisting of about 300 individuals, who underwent PET and cerebrospinal fluid collection if determined to have mild cognitive impairment (MCI) or greater. Individuals underwent follow-up analyses to track the progression of disease. People included in the study consist of those diagnosed with AD dementia (ADD), non-AD dementias (non-ADD), MCI, or subjective memory complaints (SMC).

#### PPMI

The Parkinson’s Progression Markers Initiative (PPMI) is a large study focused on the investigation of biological markers of Parkinson’s disease (PD). Founded in 2010 with support from the Michael J. Fox Foundation, PPMI plans to enroll about 4,000 people in intensive clinical and imaging testing and about 50,000 more for genotyping and simpler PD-related tests. Individuals undergo cerebrospinal fluid draws, MRI, motor assessments, and many more tests to get a comprehensive view of PD-related phenotypes. For more details on PPMI, please visit www.ppmi-info.org.

### Cerebrospinal Fluid Sample Collection

Cerebrospinal fluid (CSF) samples were collected through lumbar puncture from participants after an overnight fast. Samples were processed and stored at −80 C until they were sent for protein measurement. In total, 4,223 CSF samples were collected from unique participants. Details separated by cohort are found in Supplementary Table S1.

### Proteomic Data Processing

CSF samples from ADNI, DIAN, MAP, Ace Alzheimer Center Barcelona (FACE), and Barcelona-1 were sent for protein measurement using the SOMAscan platform (SOMAscan7k, Supp. Table 2)^24^, measuring 7,584 unique aptamers corresponding to 6,179 unique human protein targets. CSF samples from PPMI were sent for protein measurement on an older version of the SOMAscan platform (SOMAscan5k, Supp. Table 3)^24^, measuring 4,785 unique aptamers corresponding to 4,131 unique protein targets. Both panels reported aptamer levels in relative fluorescent units (RFU).

The SOMAscan7k data underwent initial normalization by SomaLogic. At the sample level, they performed hybridization normalization. The aptamers were then divided into S1, S2, and S3 normalization groups based on signal to noise ratio. SomaLogic then performed median normalization to remove biases due to protein concentration, pipetting variation, reagent concentration variation, and assay timing among others^138^. Each sample was normalized to a reference to account for technical and biological variance. This step was performed with iterative Adaptive Normalization by Maximum Likelihood (ANML) until convergence was reached. This method is a modification of median normalization. Additional normalization procedure details are documented in Somalogic’s technical note^24^.

Further quality control was performed on the normalized SOMAscan7k data provided by SomaLogic according to an in-house protocol. Aptamers were removed if they failed either of two criteria: first, if the maximum absolute difference between aptamer scale factor and median scale factor of any plate is > 0.5; second, if the median cross-plate coefficient of variation (CV) was > 0.15. Interquartile range (IQR) was then calculated for every aptamer based on log-10 transformed aptamer levels. Aptamer values outside of 1.5-fold of the IQR were replaced with NA values. Aptamers with call rate < 65% (aptamer measurement in less than 65% of samples) were excluded, and the same criteria was used to remove samples. Call rate for aptamers was then recalculated and a more stringent call rate threshold of 85% was applied. Sample call rate was recalculated after aptamer removal and a call rate threshold of 85% was applied at the sample level. A summary of the QC steps and aptamer/samples removed at each step is found in supplementary Fig. 1A. A summary of the samples removed by cohort is found in Supplementary Table S4.

Quality control was also performed on the SOMAscan5k data. Because information relating to scale factor and CV were not available for the PPMI cohort, those filtering steps were not performed. IQR-based and call-rate based quality control were performed using the same methods as with the SOMAscan7k dataset. A summary of the QC steps for the SOMAscan5k dataset is found in supplementary Fig. 1B.

To determine the number of unique proteins in each dataset (discovery and replication), we used information provided by Somalogic to map the aptamer ID to a Uniprot ID. If the aptamer was present in both the SOMAscan 5k and 7k datasets, we prioritized the mapping in 7k. We then determined the number of unique Uniprot IDs included in each dataset after QC.

### Genomic Data QC

All proteomics samples were matched to genomic data (if available) pre-proteomics QC. For ADNI, 756/758 samples also had genomic data; for MAP, 944/948; for DIAN, 421/495; for FACE^137^, 460/632; for Barcelona-1, 227/232; for PPMI, 918/1158. Genomic datasets from ADNI, MAP, DIAN, FACE, and Barcelona-1 were genotyped on multiple different arrays at different times and were imputed individually using the GRCh38 Version R2 reference panel on the TOPMed imputation server. Before imputation, high-quality directly sequenced variants were filtered based on the following criteria: (1) genotyping rate ≥ 98% per SNP or individual; (2) MAF ≥ 0.01; and (3) Hardy-Weinberg Equilibrium (HWE) P ≥ 1×10^− 6^. The datasets were then merged and variants with genotyping call rate ≥ 90%, minor allele count (MAC) ≥ 10, and Hardy-Weinberg Equilibrium (HWE) *P* ≥ 1X10^− 6^ were kept. Finally, ambiguous SNPs were removed from the analyses to avoid false-positive results due to genotyping array strand differences.

Genomic samples were first matched to the cleaned proteomic dataset, decreasing the number of samples from 3,726 to 3,581. Principal component analysis (PCA) was performed on the 3,581 samples using PLINK1.9^139^ to account for population stratification. 1000 Genome Project samples were included and used as an anchor for ancestry grouping and outlier removal. Samples that were not genetically part of the non-Hispanic White (NHW) population were first filtered using a broad criteria of gPC1 < .005 and gPC2 >−0.01 (Supplementary Fig. 2A). Further filtering was done by removing samples that were outside of three standard deviations from the mean of the remaining samples. After PCA, 3,328 NHW samples remained (a decrease of 253, Supp. Table 4). Checks for cryptic relatedness through identity by descent (IBD) were performed using PLINK1.9^139^ including only variants with MAF ≥ 0.15 or HWE P ≥ 0.001 and R^2^ of 0.2 (Supplementary Fig. 2B). Pairs with PI_HAT value ≥ 0.25 were considered cryptic relatedness and one sample from that pair was removed to minimize samples lost (N = 221). After sample removal based on cryptic relatedness, 3,107 samples remained and were used for. A summary of the samples removed per cohort in each step is available in supplementary table 3.

### pQTL Identification

We used a three-stage analysis approach (discovery, replication, and meta-analysis) to identify protein quantitative trait loci (Fig. 1). The discovery cohort consisted of ADNI, FACE, and PPMI for a total of 1,912 samples after QC. Because protein levels for PPMI were measured using SOMAscan5k, approximately 2,700 proteins included in the SOMAscan7k platform were only measured in ADNI and FACE; those aptamers were limited to a sample size of 1,127 for the discovery cohort. The replication cohort consisted of 1,195 samples from Barcelona-1, DIAN, and MAP after QC. All of these were measured on the SOMAscan7k platform.

pQTL analyses in both the discovery and replication cohorts were performed using PLINK2^139^. Aptamer levels were z-score normalized by first transforming to log10-scale and then normalizing using the scale() function in R with options scale and center set to true, to center the values around zero with a variance of 1. For both analyses, age, self-reported sex, the first ten genetic principal components, and cohortArray (for example ADNI_OmniEx, coded as a dummy variable) were used as covariates for an additive linear model in PLINK2^139^. The overall model was as follows:

$$Zscore\left(aptamer\;level\right)∼\beta_{0}+\beta_{1}*SNP\;dosage+\beta_{2}*age+\beta_{3}*sex+\sum_{j}^{4-13}\beta_{j}*gPC1-gPC10+\sum_{k}^{14-n}\beta k*cohortArray+\varepsilon$$

*n* in the equation above differs between discovery and replication due to differences in the number of cohortArray dummy variables included as covariates.

### *Cis*-pQTL identification

We defined *cis*-pQTL as all variant-aptamer associations with raw *P* < 5×10^− 8^ that were within 1MB in either direction of the transcription start site of the corresponding protein-coding gene based on hg38 coordinates (Fig. 1).

### *Trans*-pQTL identification

To define *trans*-pQTL, we calculated the multiple testing correction as follows. We calculated the number of proteomics principal components necessary to account for 95% of the variance in protein level between individuals, based only on the samples measured on the SOMAscan7k platform (I.e., excluding all samples from PPMI) to ensure as many proteins were present as possible. In total 1,450 proteomic PCs were necessary to account for that variance, so a study-wide significant p-value of 5×10^− 8^/1450 (approximately P < 3.45×10^− 11^) was used to define *trans*-pQTL. We considered all study-wide significant variant-aptamer associations farther than 1MB in either direction from the transcription start site to be *trans*-pQTL (Fig. 1).

### Meta-analysis

We utilized METAL^140^ to perform a fixed-effect meta-analysis using inverse-variance weighting for each aptamer. To correct for any remaining population stratification and relatedness, we included the option GENOMICCONTROL. To identify significant pQTL (either cis or trans) in the meta-analysis, we required a variant-aptamer association to reach *P* < 0.005 in discovery, *P* < 0.05 in replication, to have the same direction of effect in discovery and replication, and to have reached the study-wide thresholds (P < 5×10^− 8^ for cis and P < 3.45×10^− 11^ for trans) in the meta-analysis (Fig. 1).

### Identification of associations

We identified index variants used to define a pQTL through a distance-based method. For each aptamer, we scanned the genome to identify the single most significant variant-aptamer association that passed the study-wide multiple testing correction criteria and defined it as the index variant for that association. We then grouped all significant variant-aptamer associations within 1MB of the index variant and considered them the same signal. After excluding the variants in that region, we performed the same procedure for the next most significant variant-aptamer association (if applicable) until no associations reached the threshold for study-wide significance.

### Disease-specific analyses

Using PLINK2^139^, we also performed a joint association analysis utilizing all samples from discovery and replication, focusing only on the aptamers with a significant association in the meta-analysis and the top variant-aptamer pairs that made up those associations. We considered age, sex, genetic principal components 1–10, and cohortArray as covariates in the model. We used Alzheimer’s disease-specific biomarkers amyloid beta 42 (Aβ42) and phosphorylated tau-181 (pTau, both measured in CSF) to determine amyloid/tau classification^25^ for each sample with both measurements. To determine classification of each sample by amyloid and tau positivity, we performed dichotomization using the mclust R package^141^. Dichotomization was performed separately for each cohort (ADNI, DIAN, MAP, Barcelona-1, and FACE; PPMI was not analyzed because amyloid and tau measurements were not available). Aβ42 and pTau levels were then log10-transformed to approximate a normal distribution and were normalized with z-score transformation to have a mean 0 and standard deviation 1. Outliers were removed based on a cutoff of 3*1SD from the mean. Standardization by z-score was recalculated after outlier removal.

For MAP, the LumiPulse G platform (Fujirebio US, Inc, Malvern, PA) was used to measure both Aβ42 and pTau. For seventeen samples with missing LumiPulse values, we used Innotest (Fujirebio) values instead due to high correlation between the two platforms (r = 0.73 for AB42 and r = 0.86 for pTau). Dichotomization was performed for 948 total subjects for CSF Aβ42 and 944 for CSF pTau. A cutoff of z-score = −0.20 was obtained for Aβ42 corresponding to a raw value of 630 pg/mL. Samples below 630 were considered Aβ42 positive. A cutoff of z-score = 0.61 was obtained for pTau corresponding to a raw value of 62.9. Samples above 62.9 were considered pTau positive.

For ADNI, Innotest (Fujirebio) was used for Aβ42 and Elecsys (F. Hoffmann-La Roche Ltd, Switzerland) for pTau. Dichotomization was performed for 749 subjects for AB42 and 745 for pTau. A z-score cutoff of 0.616 was identified for AB42, corresponding to a raw value of 196 pg/mL. Samples below 196 were considered AB42-positive. A z-score cutoff of 0.197 was identified for pTau, corresponding to a raw value of 27.8. Samples above 27.8 were considered to be pTau-positive.

For Barcelona-1, Innotest (Fujirebio) was used for both Aβ42 and pTau. Both analyses included 231 samples. A z-score cutoff of 1.04 was identified for Aβ42, corresponding to a raw Aβ42 value of 1325 pg/mL. Samples below 1325 pg/mL were considered Aβ42-positive. A z-score cutoff of −0.163 was identified for pTau, corresponding to a raw value of 58. Samples above 58 were considered to be pTau-positive.

For FACE^142^, Innotest (Fujirebio) was used for both Aβ42 and pTau. Both analyses included 632 samples. A z-score cutoff of 0.468 was identified for Aβ42, corresponding to a raw Aβ42 value of 856 pg/mL.

Samples below 856 were considered Aβ42-positive. A z-score cutoff of −0.018 was identified for pTau, corresponding to a raw value of 67. Samples with a value greater than 67 were considered pTau-positive.

For DIAN, LumiPulse (Fujirebio) was used for both Aβ42 and pTau. Dichotomization for Aβ42 was performed on 478 samples and 474 for pTau. Five Aβ42 values and ten pTau values were replaced with CSF_xMAP (MilliporeSigma, Burlington, MA) platform measurements due to missingness (r = 0.77 and 0.89 between platforms for Aβ42 and pTau respectively). A z-score cutoff of −0.198 was identified for Aβ42, corresponding to a raw Aβ42 value of 517 pg/mL. Samples below 517 were considered to be Aβ42-positive. A z-score cutoff of 0.31 was identified for pTau, corresponding to a raw value of 51.8. Samples with a value greater than 51.8 were considered pTau-positive.

Samples with proteomic data were then matched to samples that had genomic data that passed quality control. Amyloid and tau-positive (A^+^T^+^, 775) samples were treated as cases and amyloid and tau-negative samples (A^−^T^−^, 889) were treated as controls. We stratified our samples by biomarker status and used PLINK2 with age, sex, genetic PCs 1–10, and cohortArray to test A^+^T^+^ samples and A^−^T^−^ samples separately for association of the index SNPs identified in the meta-analysis with their corresponding aptamer levels. We then correlated effect size between the meta-analysis and each classification as well as comparing them to each other (supplementary Fig. 7 and supplementary table X).

### Conditional Analysis

We utilized GCTA-COJO^26,27^ to perform approximate conditional analysis on our identified pQTL. For each variant-aptamer association, we converted the summary statistics from METAL to the format readable by COJO and performed conditional analysis on *cis* and *trans* associations using the appropriate p-value threshold.

### Variant Annotation

We utilized the Variant Effect Predictor (VEP)^28^ to annotate the most severe consequence for each of our pQTL associations. For each association, we identified all variants in high LD (*R^2^* > 0.8) with the index pQTL variant in that association. We then used those as input to VEP and obtained all annotation information for each variant. We then grouped each set of correlated variants and determined the most severe annotation for the association based on VEP’s order of severity.

### Enrichment of annotation categories

To test for enrichment of the variant annotation categories as identified by VEP, we first determined the most severe annotation based on the methodology above for all independent variants. We then used a permutation-based approach where we randomly selected a group of 3,373 variants from all those assayed. Using the annotation approach above, we determined the most severe annotation for each of the randomly selected variants. We repeated this 1,000 times and obtained a distribution of the number of times each annotation was found in each group of 3,373 randomly-selected variants (I.e. for one permutation, 500 out of 3,373 were annotated as missense variants). To get a p-value, we then compared our actual independent pQTL variants to the randomly generated distribution and determined the number of permutations where more variants were mapped to each variant type than the actual data. If the pQTL variants had more of a variant type than any of the random sets, the p-value was set at P < 0.0001 (1/1000).

### Identification of Drug Targets

Using the Uniprot^143^ IDs corresponding to each aptamer as supplied by Somalogic (see Supp. Table S2 & S3), we queried the Uniprot^143^ database “DrugBank” entry using their advanced search features. We then matched the DrugBank^110^ molecule IDs to each of the corresponding aptamers (Supp. Table S5).

### External Replication

We utilized an external dataset supplied by the Stanford Alzheimer’s Disease Research Center (SADRC) and Aging Memory Study (SAMS) that included whole genome sequencing data as well as CSF proteomic data measured using either the SOMAscan5k assay^24^.

### Stanford Iqbal Farrukh and Asad Jamal ADRC

Samples were acquired through the National Institute on Aging (NIA)-funded Stanford Alzheimer’s Disease Research Center (SADRC). The SADRC cohort is a longitudinal observational study of clinical dementia subjects and age-sex-matched non-demented subjects. The collection of plasma was approved by the Institutional Review Board of Stanford University and written consent was obtained from all subjects. Blood collection and processing were done according to a rigorous standardized protocol to minimize variation associated with blood draw and blood processing. Briefly, about 10 cc whole blood was collected in a vacutainer EDTA tube (BD Vacutainer EDTA tube) and spun at 3000RPM for 10 mins to separate out plasma, leaving 1 cm of plasma above the buffy coat and taking care not to disturb the buffy coat to circumvent cell contamination. Plasma processing times averaged approximately one hour from the time of the blood draw to the time of freezing and storage. All blood draws were done in the morning to minimize the impact of circadian rhythm on protein concentrations.

All healthy control participants were deemed cognitively unimpaired during a clinical consensus conference that included board-certified neurologists and neuropsychologists. Cognitively impaired subjects underwent Clinical Dementia Rating and standardized neurological and neuropsychological assessments to determine cognitive and diagnostic status, including procedures of the National Alzheimer’s Coordinating Center (https://naccdata.org/). All participants included in this study were deemed cognitively impaired during a clinical consensus conference that included neurologists and neuropsychologists. All participants were free from acute infectious diseases and in good physical condition.

### Stanford Aging Memory Study (SAMS)

SAMS is an ongoing longitudinal study of healthy aging. Blood collection and processing were done by the same team and using the same protocol as in SADRC. Neurological and neuropsychological assessment were done by the same team and using the same protocol as in SADRC. 192 participants were included in the present study, and 11 were participants in both the SADRC and SAMS study.

### Genomic/proteomic data QC

We performed genomic principal component analysis and identity by descent filtering based on the genomic data, using 1000 Genomes samples as an anchor, based on the same general steps as for the main analysis. Plink1.9^139^ was used for both filtering steps. The whole genome sequencing data was filtered using a genotyping rate threshold of 95% and a HWE threshold of 5×10^− 8^. After PCA filtering on 582 samples to select non-Hispanic white individuals, 478 samples remained. After IBD filtering based on PI_HAT of 0.2, 460 samples remained. As with the genomics data, equivalent quality control steps were used to filter the SOMAscan5k-based proteomics data as well. Starting from 283 samples with proteomics data, filtering based on scale factor, coefficient of variation, interquartile range, and sample/individual call rate removed nine samples and 260 aptamers, leaving 274 samples and 4,735 aptamers remaining. After matching between proteomics and genomics samples, 183 were used for pQTL analysis using the SOMAscan5k platform.

### Tissue-specificity of pQTL Associations

To determine the overlap of significant CSF pQTL associations across tissues, we compared the pQTL associations from our meta-analysis to those identified in the largest-to-date aptamer-based plasma pQTL study^11^ and to plasma and brain pQTL^16^. We performed Bayesian colocalization using coloc.abf^58^. We used a region of 1MB surrounding the index CSF pQTL variants to select variants for inclusion in the analysis. We included only variants that were present in both analyses. We tested for a shared genetic association between our CSF pQTL signals and both plasma datasets and the brain dataset, ensuring that the aptamer ID was consistent between each dataset analyzed. We considered an association to be shared between tissues according to colocalization if the posterior probability of H4 (shared association) was greater than or equal to 0.80. The plasma and brain analyses published by Yang et al. were based on the GRCh37 genome build, so we lifted over the summary statistics to GRCh38 coordinates using the UCSC Genome Browser’s LiftOver tool^144^. Due to limited aptamer overlap, we were able to perform colocalization for 1,682 associations with Ferkingstad et al.^11^, 389 for in-house plasma^10^, and 465 for in-house brain^10^ (out of 2,316). We determined if *trans* associations were more likely to be CSF-specific than *cis* using the prop.test() function in R^145^. The remaining 634 associations could not be analyzed because the corresponding aptamer was not measured in the plasma study.

### Molecule-specificity of pQTL associations

To determine the overlap between protein QTL associations and RNA transcript QTL (eQTL) associations, we analyzed whole blood eQTL summary statistics from eQTLGen^5^ and GTEx^4^, cortex eQTL from GTEx, microglia-specific eQTL^29^ and cortex, hippocampus, basal ganglia, cerebellum, and spinal cord eQTL from MetaBrain^6^. The summary statistics from eQTLGen were supplied using GRCh37, so we lifted them to GRCh38 coordinates using LiftOver^144^. For the eQTL datasets, if Ensembl transcript ID was supplied, we matched using only the Ensembl gene ID and ignored the transcript info. Ensembl ID was matched to the Entrez Gene Symbol using the biomaRt R package^146^, then the Entrez Gene Symbols for each protein aptamer were used to match summary statistics between pQTL and eQTL datasets. Because most of these datasets were limited to *cis*-acting variants only, we performed colocalization using significant *cis* pQTL associations only. For each pQTL association, we kept all SNPs present in both pQTL and eQTL datasets that were within 1MB of the index pQTL variant’s location. We utilized the coloc.abf function^58^ to perform Bayesian colocalization. We determined shared associations between pQTL and eQTL to be those with PP.H4 ≥ 0.80.

### Identification and Analysis of Pleiotropic Regions

We utilized an LD-based method to define pleiotropy. For each index pQTL variant identified, we used PLINK1.9^139^ to determine all other index pQTL variants in LD (*R^2^* > 0.1). We then grouped all sets of variants that were in LD with each other and defined the region as all base pairs between the two farthest-apart index variants of each LD group. We then grouped all aptamers with a pQTL into one of these regions based on the association’s index variant and determined the number of aptamers and number of corresponding Entrez Gene Symbols whose index variants were located in each pQTL region. Because of the complexity of the human leukocyte antigen (HLA) region located on chromosome six, we manually curated this region to encompass all index variants either from the start of the HIST1H2AA gene to the end of the RPL12P1 gene or in LD (*R^2^* ≥ 0.1) with either of those variants, producing a pQTL region spanning from chr6:25317524 to chr6:33304176^41^. We then performed further analysis on the three regions with the highest number of associated proteins.

For each of the three highly pleiotropic regions (chr3:190886452–190950415, chr6:25317524–33304176, and chr19:44888997–44919689), we first determined the localization of the gene encoding each regulated protein. We matched each gene to a corresponding brain-relevant cell type (see Cell-Type Specificity Analysis). Using the circlize R package^147^, we obtained circos plots showing the associations for each region. Next, using the gene IDs as determined by Somalogic, we performed pathway analysis for the proteins regulated by each pleiotropic region (see Pathway Enrichment Analysis) to determine the biological mechanisms regulated by the regions. We performed cell-type enrichment analysis (see below) to determine if the pathways were especially relevant to a specific cell type. Finally, we utilized the GWAS Catalog^30^ to perform a phenome-wide association study (pheWAS). For each of the three regions, we used the index pQTL variants identified in each and queried the GWAS Catalog to identify all genome-wide significant associations for those variants. This was done to elucidate diseases and traits that may be regulated by the region and may include regulated proteins in their etiology. We performed LD pruning using PLINK2^139^ using a window size of 500KB, step size of 50, and *R^2^* cutoff of 0.5 to define independent LD blocks. We utilized Haploview^148^ to visualize the linkage disequilibrium structure of these pleiotropic regions.

We compared the number of aptamers associated with each region in CSF and plasma^11^. Using the index associations identified in plasma (N = 18,084), we determined the number of plasma associations in each of the three main CSF pleiotropic regions. Using the total number of index associations identified (2,316 in CSF and 18,084 in plasma), we performed a two-sided two-sample z-test for proportions using prop.test() in R to analyze the difference in the proportion of the total associations between tissues in each region.

Because the *APOE* chr19 region is so intertwined with disease, we sought to determine if the associations seen were driven by disease status, To do this, we tested for a difference in effect size between AT^−^ and AT^+^ individuals for each of the associations in this region using the following equation:

$$Zdiff=\frac{\beta_{AT^{-}}-\beta_{AT^{+}}}{\sqrt{SE_{AT{-}^{2}}+SE_{AT{+}^{2}}}}$$

We then used a Z-statistic cutoff of ±1.960 (corresponding to P = 0.05) to determine associations whose strength differed between AT^−^ and AT^+^ samples.

### Alzheimer’s Disease Proteome-Wide Association Study

To identify proteins potentially involved in the etiology of AD, we utilized a modified version of the FUSION framework^55^. In brief, FUSION calculates the genetic contribution of each SNP in a region of interest to the level of an aptamer. Then, by matching the variants to those in a binary trait GWAS, the framework uses the calculated contributions (weights) to impute protein level based on the summary statistics from the GWAS. It then correlates the genetically regulated protein levels with disease status to identify both association and directionality between protein levels and the binary trait.

For our analyses, we used the Stage 1 summary statistics consisting of 39,106 clinically diagnosed AD cases, 46,828 proxy AD cases, and 401,577 controls from the largest-to-date AD GWAS study^22^. Typically, the FUSION framework focuses only on cis-regulated abundance, utilizing the transcription start site of the gene of interest to determine the region to use for weight calculation. However, because of the substantial presence of trans-pQTL associations identified in our analysis, we instead used the index variant for each pQTL association as the reference to choose variants. We then included all variants in a 1MB region surrounding each index variant. We included the methods “top1”, “lasso”, and “enet” for weight calculations. Because we only calculated weights for regions/aptamers that had a significant pQTL, we set the heritability p-value threshold to 1 to ensure that all association/aptamer pairs were analyzed for association. We included age, self-reported sex, genetic principal components 1–10, and cohortArray as covariates in the weight calculation model, matching those used for the initial pQTL identification. We then prepared a file containing information about the calculated weight files for each pQTL for input to the association analysis step.

To calculate association between imputed protein levels and AD status, we ran FUSION.assoc_test.R on each autosomal chromosome, using the same binary file used to calculate the weights further subsetted by chromosome. This calculates imputed protein levels in the AD GWAS summary statistics based on the weight files calculated in the previous step. It then associates the imputed protein levels with disease status. We used Benjamini-Hochberg^149^ false discovery rate correction with an adjusted threshold of *P*_*FDR*_ < 0.05 for association between each variant-aptamer pQTL region (because we are including both *cis* and *trans* associations, we cannot specify just aptamer) and disease status to determine significant aptamer-disease associations.

### Alzheimer’s Disease Colocalization Analysis

#### Colocalization under a single causal variant assumption

To determine shared genetic etiology between aptamers and AD, we performed Bayesian colocalization analysis using coloc.abf from the coloc R package^58^. We used the default prior probabilities of *P*_1_ = 1x10^− 4^, *P*_2_ = 1x10^− 4^ and *P*_12_ = 1x10^− 5^. For a genetic signal to be considered shared between aptamer level and AD status, we required that the posterior probability of H4 (PP.H4) be > 0.8. This threshold suggests high confidence that the genetic signals for the two traits are caused by the same variant. We used the same AD GWAS as for FUSION^22^. We included all variants within 1MB of the pQTL index variant that were present in both our pQTL analysis and the AD GWAS summary statistics. For each of these, we harmonized the effect allele to ensure consistent direction of effect between the two analyses. Using PLINK1.9^139^, we calculated an LD matrix based on the 3,107 samples analyzed. While the matrix is not necessary for this method, for consistency with COLOC-SuSiE we used it to filter all variants missing LD info before running colocalization. This ensured the same variants were used in both analyses. Because both the pQTL analyses and AD GWAS report the standard error of the effect size for each variant, we squared the standard error for each variant in each analysis to obtain the variance as required for coloc. Using only the variants that were present in both analyses, colocalization was then performed. For the AD GWAS, the “type” was set to “cc”. Because z-score protein levels were used, “sdY” was set to 1 for the pQTL dataset.

#### Colocalization with no single causal variant assumption

To relax the single causal variant assumption inherent in coloc^58^, we also performed colocalization using coloc-SuSiE, which accounted for LD and was able to compare multiple independently significant variants at each locus^59^. This approach first utilizes SuSiE^150^, which uses an iterative Bayesian stepwise selection approach to identify independent credible sets of variants, each of which contains a variant predicted to be causal. This is performed on both the pQTL data and the AD GWAS data^22^, to get two separate lists of credible sets for each trait. The same variants as above were used for colocalization, which is then run on each pairing of credible sets. The LD matrix and the effect size and variance for each variant was then supplied to the runsusie function in the coloc R package, which calculated the credible sets for each trait. This information was then passed to the coloc.susie function in the coloc R package to calculate colocalization. A threshold of PP.H4 > 0.8 was used to identify shared causal variants.

#### Alzheimer’s Disease Mendelian Randomization

In order to estimate causality of the proteins measured by the aptamers in this analysis, we implemented MR using the R package TwoSampleMR^57^. TwoSampleMR first performs LD-based clumping to identify independent instrument variables (variants) for each exposure (aptamer). We performed clumping on each aptamer with a study-wide significant pQTL. We limited the variants included for each aptamer to only those that reached genome-wide significance (*P* < 5x10^− 8^) for that aptamer, using the predefined default clumping thresholds. We extracted the instrument variants identified by clumping then harmonized the two datasets to ensure matching effect alleles. To account for pleiotropy in the analysis, we first removed all *trans*-associated variants for each aptamer including any variant in a pleiotropic region (as identified above) that regulated five or more aptamers. We then further removed all *trans*-associated variants within 500KB of rs429358 (*APOE* ε4) due to the highly significant association of this region with AD. Because *cis*-regulated proteins in general are higher confidence to be causal than *trans*, we did not remove any *cis* variants based on pleiotropy. *Cis* instrument variables that are located in pleiotropic regions (regions regulating five or more aptamers) are marked in Supp. Table S46. We then performed MR analysis to determine causality of all aptamers with a pQTL for AD risk. For all analyses with one instrumental variable, we utilized the Wald Ratio p-value. For all analyses with two or more instrumental variables, we utilized the inverse variance weighted (IVW) p-value. To view the results from other methods, see Supp. Table S34. We performed multiple test correction using Benjamini-Hochberg^149^ false discovery rate, with an adjusted p-value threshold of 0.05.

### Proteomic Risk Scores

#### AT status prediction using all PWAS aptamers

Proteomic risk score analysis^116^ was performed to predict amyloid/tau status of our samples^25^, based on the dichotomization of samples discussed above (see Disease-specific analysis). We stratified our samples to only those considered amyloid and tau positive or amyloid and tau negative, excluding all samples with missing information or that were positive for one and negative for the other. To calculate the values used for the prediction model, we first limited our matrix to only aptamer that had a significant association with AD as identified by PWAS (456 aptamers). If the PWAS-reported effect size for an aptamer was negative (i.e., higher levels of the aptamer were protective against AD), the z-score level of that aptamer was flipped in each individual, so negative values became positive and vice versa. This was done to ensure higher aptamer levels were always associated with increased AD risk according to PWAS. For each sample, the sum of the z-score levels for the 456 aptamers was calculated. Because each sample has different levels of missingness, the sum was then divided by the number of non-missing aptamer levels out of 456.

We first determined the difference in proteomic risk score value distribution between unrelated A^−^T^−^, A^+^T^−^, and A^+^T^+^ samples (N = 785, 496, and 733, respectively) using the Wilcoxon rank-sum test^151^ as implemented in the compare_means function in the ggpubr R library. A^−^T^+^ samples were included because of unclear relevance to AD. Samples from DIAN were excluded due to the high prevalence of early-onset forms of disease while the proteins were chosen based on late-onset AD.

#### AT status prediction using aptamers chosen by LASSO regression

We utilized the R package “glmnet” to perform LASSO regression^152^ to identify significant predictors of AT status. Because this method requires that there be no missing values, we first imputed the values by separating the samples into A^+^T^+^, A^+^T^−^, and A^−^T^−^ groups. For each sample in each group, we identified the aptamers with missing values and replaced the missing values with a value randomly sampled from the corresponding aptamer levels of the other samples in that group (with the seed set to one for consistency). We split our samples into training and testing datasets matching the discovery and replication used for QTL analysis (training: ADNI and FACE, N = 828; testing: MAP & Barcelona-1, N = 690). We then used cv.glmnet (with the seed set to one for consistency) to calculate lambda values for each number of aptamers in the training data. The weights from the model with the lowest lambda were then used to predict AT status in the training and testing datasets. The best model (lowest lambda) identified 162 aptamers with nonzero weights (Supp. Table S31).

We also performed age and *APOE* genotype-stratified analyses using the LASSO-defined model. As with the training and testing datasets, we used the predict() command to obtain values for each individual that were then used to predict AT status. The stratified datasets were obtained by selecting all samples fitting the stratification criteria (age bracket or *APOE* genotype) from both the testing and training datasets.

#### Risk prediction using polygenic risk scores

Because the proteins prioritized for use in the proteomic risk scores were identified based on genetic associations, we wished to compare the predictive ability of the ProtRS vs a polygenic risk score (PRS) calculated using variants associated with AD status^22^. The PRS for each individual was calculated using a p-value threshold of P < 5x10^− 8^. PRSice2^153^ using default clumping parameters (--clump-p 1, --clump-r2 0.1, --clump-kb 250) was used to calculate the PRS using --score std. For each sample, a PRS was calculated both including and excluding the APOE region, as defined as chr19:43907927–45908810 in GRCh38. The PRS including *APOE* was used for all analyses except the *APOE*-stratified analysis, where the PRS excluding *APOE* was used. For the training and testing datsets, we fit a generalized linear model where the PRS value per individual, age, and sex were used as inputs and AT status was defined as the outcome. The GLM was then used to predict AT status using the predict() command. For age-stratified analyses, the PRS and sex were included in the model; for *APOE*-stratified, the PRS, age, and sex were included. For all models, confidence intervals and p-values between ProtRS and PRS were obtained using the ci.auc and roc.test functions in the pROC library^154^.

#### External replication of proteomic risk scores

Because we developed and tested our model in samples that were measured for proteomics and processed at the same time, we sought to validate our findings in an external dataset (Stanford ADRC) that was assayed separately. We predicted A/T status using the model defined in the training dataset. Because the proteomics measurements were based on the SOMAscan5k platform, some proteins included in the model were not measured (288/456 measured, 106/162 significant predictors measured). Therefore, all missing measurements were replaced with zero to remove their effects on the model. As with our training and testing datasets, we first limited the dataset to unrelated, NHW samples as determined by IBD and PCA. We then used the training model to estimate a risk value for each individual (58 A−T−, 21 A+ T+) based on the non-missing aptamers. This was compared to a GLM using only age and sex as predictors for reference. CSF Aβ42, Aβ40, total tau, and p-tau181 were measured using the fully-automated Lumipulse G1200 platform (Fujirebio US, Inc, Malvern, PA). Amyloid-positivity was assigned to CSF samples with an Aβ42/Aβ40 ratio below 0.091, while tau-positivity was assigned to samples with total tau > 556.43 pg/ml and/or p-tau181 > 75.273 pg/ml. Optimal cutpoints were determined using the Youden method to optimize sensitivity and specificity as previously described (CITE https://alzres.biomedcentral.com/articles/10.1186/s13195-022-01116-2).

#### Pathway Enrichment Analysis

We performed pathway enrichment analysis for multiple groups of aptamers. We performed pathway analysis in R using enrichGO, enrichDGN, enrichKEGG, gseGO, Reactome, and enrichDO. EnrichGO, enrichKEGG, and gseGO were implemented using the clusterProfiler package from Bioconductor^155^. EnrichDGN and enrichDO were implemented using the DOSE package from Bioconductor^156^. Reactome was implemented using the ReactomePA package from Bioconductor^157^. For each set of aptamers, we used the annotation file supplied by SomaLogic to map the aptamers to an EntrezGeneSymbol. We did the same for all aptamers in the panel. For enrichGO, enrichDGN, enrichKEGG, Reactome, and enrichDO, we supplied the list of selected Entrez gene symbols as input (IDs instead of symbols for enrichDGN) and set the universe to all unique genes covered by the SOMAscan7k panel (n = 6,112). For gseGO, the default universe was used, as there is not a command to change the background. For all enrichment analyses, an FDR-corrected p-value threshold of 0.05 was used.

#### Cell-type Specificity Analysis

We downloaded gene expression data from human astrocytes, neurons, oligodendrocytes, microglia/macrophages, and endothelial cells^34^ to determine the degree of specificity to relevant cell types for the aptamers included in the SOMAscan7k panel. The data downloaded contained multiple subtypes of astrocytes; we focused only on human mature astrocytes for our analysis. For each cell type, we determined the average expression level across individuals for each gene. We then added the averages from each cell type to get a total expression level for that gene across the five cell types. We then calculated the percentage of the total expression that each cell type contributed. A gene was reported to be cell-type specific if the percentage of its full expression contributed by the top cell type was 1.5x higher than the second top cell type. For example, 72.5% of the brain expression of *APOE* was attributed to mature astrocytes, while the next highest cell type was neurons at 10.6%. Because the ratio of astrocyte contribution (72.5%) to neuron contribution (10.6%) is greater than 1.5, *APOE* was considered astrocyte-specific.

To determine enrichment, we first matched each of the proteins in the SOMAscan7k platform to their EntrezGeneSymbols using the Somalogic-provided documentation. Using the above ratio-based strategy, we determined the cell-type specificity for all EntrezGeneSymbols and counted the number of genes that were specific to each cell type. In total, 5,750 proteins had corresponding genes that were included in the cell-type expression data. For each protein subset, we determined the number of corresponding genes that were specific to each cell type. We then tested for enrichment using the hypergeometric test in Excel.

## Figures and Tables

**Table 1:** Demographics of post-QC samples used in discovery and replication.

Cohort	# Proteomics Samples	Avg. Age	%Male	%APOE4+	#CO	#AD	#ADAD	#ADRD	#PD
Discovery									
ADNI	689	73.7 (SD 7.5)	58.2	50.2	149	521	0	19	0
PPMI	785	61.8 (SD 9.4)	55.1	NA	157	0	0	1	627
FACE	438	71.9 (SD 8.3)	41.1	35.6	128	238	0	72	0
Replication									
DIAN	193	38.6 (SD 10.7)	48.4	27.6	73	3	116	1	0
MAP	805	71.4 (SD 8.7)	46.7	39	565	176	2	59	3
Barcelona-1	197	68.8 (SD 7.5)	52.3	NA	4	63	0	129	1
Total	3107				1076	1001	118	281	631

ADNI: Alzheimer’s disease Neuroimaging Initiative; PPMI: Parkinson’s Progression Markers Initiative; FACE: Ace Alzheimer Center Barcelona; DIAN: Dominantly-Inherited Alzheimer’s Network; MAP: Knight ADRC Memory and Aging Project

%APOE4+: Percentage of individuals who are carriers of at least one C allele at rs429358.

#CO: Number of cognitively normal individuals

#AD: Number of individuals affected with late-onset Alzheimer’s disease, as determined by clinical status

#ADAD: Number of individuals affected with early-onset Alzheimer’s disease

#ADRD: Number of individuals affected with non-AD dementia (including frontotemporal dementia, lewy body dementia, etc.)

#PD: Number of individuals affected with Parkinson’s disease

## Data Availability

Proteomic data from the Knight ADRC participants are available at the NIAGADS and can be accessed at https://www.niagads.org/Knight ADRC-collection; The summary results using these data are also available to the scientific community through a public web browser: www.omics.wustl.edu/proteomics. Data generated from the DIAN cohort can be requested at https://dian.wustl.edu/our-research/for-investigators/diantu-investigator-resources/dian-tu-biospecimen-request-form/. **Data used in preparation of this article were obtained from the Alzheimer’s Disease Neuroimaging Initiative (ADNI) database (adni.loni.usc.edu). As such, the investigators within the ADNI contributed to the design and implementation of ADNI and/or provided data but did not participate in analysis or writing of this report. A complete listing of ADNI investigators can be found at: http://adni.loni.usc.edu/wp-content/uploads/how_to_apply/ADNI_Acknowledgement_List.pdf
